# Pathology and parasite distribution in mice challenged with *Toxoplasma gondii* from different geographical origins

**DOI:** 10.1017/S0031182026101589

**Published:** 2026-03

**Authors:** Lauren Elaine Black, Javier Palarea-Albaladejo, Daniela Pontes Chiebao, Clare Hamilton, Paul M. Bartley, Alison Burrells, Clare Underwood, Frank Katzer, Francesca Chianini

**Affiliations:** 1 School of Psychology and Neuroscience, College of Medical, Veterinary and Life Sciences, University of Glasgow, Glasgow, UK; 2 Moredun Research Institute, Pentlands Science Park, Edinburgh, UK; 3 Department of Computer Science, Applied Mathematics and Statistics, University of Girona, Girona, Spain; 4 Biomathematics and Statistics Scotland, JCMB, The King’s Buildings, Edinburgh, UK; 5 Department of Preventive Veterinary Medicine, Faculty of Veterinary Medicine and Animal Science, University of São Paulo, São Paulo, Brazil; 6 Moredun Scientific Ltd., Penicuik, UK; 7 Labcorp, UK

**Keywords:** mice, pathology, strains, *Toxoplasma gondii*, virulence, zoonosis

## Abstract

*Toxoplasma gondii* (*T. gondii*), a zoonotic parasite, can cause severe disease in warm-blooded animals. Pathological changes in murine tissues infected with different *T. gondii* isolates were studied to establish factors influencing lesion severity and parasite burden. In Study A, mice were orally inoculated with genotype #3, #6 or #8 oocysts. In Study B, mice were inoculated intraperitoneally with genotype #1, #3, #6, #13, #141 or #265 tachyzoites. Mice were euthanised serially and tissues processed for histopathology. In Study A, genotype #6 caused pathology in the liver, brain, lung, intestine and kidney, predominantly associated with tachyzoites, while #8 caused mainly moderate pathology in the brain, lung and liver, usually associated with tissue pseudocysts/cysts. Genotype #3 occasionally caused mild pathology, but the parasite was not visible in examined tissues. In Study B, genotypes #13 and #6 caused systemic infections associated with tachyzoites. Genotypes #3, #141 and #265 caused moderate pathology associated with pseudocysts/cysts in the brain and tachyzoites in peripheral organs. Genotype #1 caused mild pathology associated with pseudocysts/cysts in organs assessed. Comparison of genotype #6 between studies showed parasite stage and inoculation method did not affect the severity of pathology, but for #3, pathology was more severe when mice were inoculated intraperitoneally with tachyzoites compared to those inoculated orally with oocysts. This study confirmed route of infection, *T. gondii* strain, life stage and dose influence infection outcome and ultimately contributes to the refinement of *T. gondii* pathogenesis knowledge, which is fundamental for toxoplasmosis management and treatment.

## Introduction

*Toxoplasma gondii* (*T. gondii*) is a protozoan parasite that causes toxoplasmosis, a widespread zoonotic disease diagnosed worldwide (Hamilton et al., 2019a) with approximately one third of the human population likely to have been exposed to *T. gondii* (Hill and Dubey, 2002). *Toxoplasma gondii* can infect most warm-blooded animals, including humans, small ruminants, rodents and birds (Buxton and Rodger, 2007; Hamilton et al., 2019a). *Toxoplasma gondii* is transmitted between hosts orally through consumption of food or water contaminated with oocysts (shed by infected cats) or by ingestion of tissue cysts found in raw or undercooked meat. Additionally, *T. gondii* can be transmitted transplacentally from mother to offspring (Hill and Dubey, 2002).

So far, at least 292 restriction fragment length polymorphism (RFLP) *Toxoplasma* genotypes have been reported worldwide (Valenzuela-Moreno et al., 2020), causing different degrees of pathology in different hosts. Strains can be separated into 2 main groups based on parasite genetics: canonical, and non-canonical (Pena et al., 2008; Chiebao et al., 2016). Currently, 12 lineages have been identified in different parts of the world, but some non-canonical strains are highly diverse with unique polymorphisms that mean they cannot be clustered and assigned to a specific lineage (Robert-Gangneux and Darde, 2012). Genotypes #1, #2 and #10 are clonal lineages and are classified as canonical (Robert-Gangneux and Darde, 2012). Genotype #10 strains are generally the most virulent canonical strains, while genotypes #1 and #2 are less virulent in mice and humans (Howe and Sibley, 1995; Su et al., 2010; Dvorakova-Hortova et al., 2014; Liu et al., 2015). Genotypes #6, #11 and #8 form the Brazilian clonal lineages and are examples of non-canonical genotypes with #6 being the most virulent, #11 being moderately virulent and most #8 isolates being avirulent or non-lethal (Pena et al., 2008). Human toxoplasmosis is generally not severe and potentially asymptomatic in the immunocompetent. In immunocompromised or congenitally infected individuals, toxoplasmosis can be severe, potentially resulting in encephalitis or death (Remington et al., 2006; Ajzenberg et al., 2009). Toxoplasmosis caused by non-canonical strains can result in severe disease that can cause death in immunocompetent individuals (Chiebao et al., 2016; da Silva Jr et al., 2017; Hamilton et al., 2019b).

In addition to *T. gondii* genotype, factors including parasite load (Saeij et al., 2005), *T. gondii* life stage (Chiebao et al., 2016; Dubey et al., 2016), host species and immune response can influence infection outcome (Gazzinelli et al., 1998; Johnson, 1998; Carruthers and Suzuki, 2007). Mice are often used to determine the virulence and disease outcome when infected with *Toxoplasma* strains as toxoplasmosis in this species often correlates with the same disease in humans (Hamilton et al., 2019a) and they show associated lesions in many organs (Pinheiro et al., 2015). The diversity of mouse virulence profiles within the same genotype has previously been shown (Chiebao et al., 2016; Calero-Bernal et al., 2022), which elicits the need for more studies associating a broader set of phenotypic traits with genotypic prediction for virulence.

The aim of this study was to characterize and compare lesions and describe parasite distribution in murine tissues infected with isolates of *T. gondii* of varying virulence to better understand the relationship between virulence and pathology.

## Materials and methods

### Tissue samples

Tissues used in this investigation were selected from the Pathology Histology Archive from the Moredun Research Institute (Edinburgh, UK) and were originally collected during 2 previously published studies that will be referred to as Study A (Chiebao et al., 2021) and Study B (Hamilton et al., 2019a). All animal procedures complied with the Animals (Scientific Procedures) Act 1986, conducted at Home Office registered premises, and were approved by the Moredun Research Institute Experimental and Ethical Review Committee (Study A: E22/14; Study B: E60/15).

Briefly, in Study A, 110 adult female Swiss-Webster mice (Hsd:ND4; Harlan Laboratories, UK) were assigned to 1 of 4 groups: the negative control group A1 (*n =* 5), A2 (*n =* 35), A3 (*n =* 35) and A4 (*n =* 35). Mice in groups A2, A3 and A4 were inoculated orally with 50 *T. gondii* oocysts (Table 1). For A1, all mice were humanely euthanised at day 21 post inoculation (p.i.). For A2, A3 and A4, 5 mice were scheduled to be humanely euthanised at days 1, 2, 3, 4, 7, 14 and 21 p.i. Mice were monitored at least 2 times daily for clinical signs of toxoplasmosis (including ruffled coat, hunched demeanour, dehydration and dyspnoea). These signs were scored and if they were above a predetermined score, mice were humanely euthanised (see Chiebao et al. (2021) for details). Brain and peripheral organs (lung, liver, kidney, diaphragm, stomach and intestine) were collected at postmortem and fixed in 10% buffered formalin.

**Table 1.** Study A groups and corresponding *T. gondii* isolates Table 1 long description.

Group number	Inoculating isolate	ToxoDB RFLP genotype	Origin of isolate	Reference
A1	–	–	–	–
A2	Moredun M4	#3	UK, Europe	Benavides et al., 2011
A3	TgCatBr71	#6	São Paulo, Brazil	Pena et al., 2006
A4	TgCatBr60	#8	São Paulo, Brazil	Pena et al., 2006

**Figure 1.**
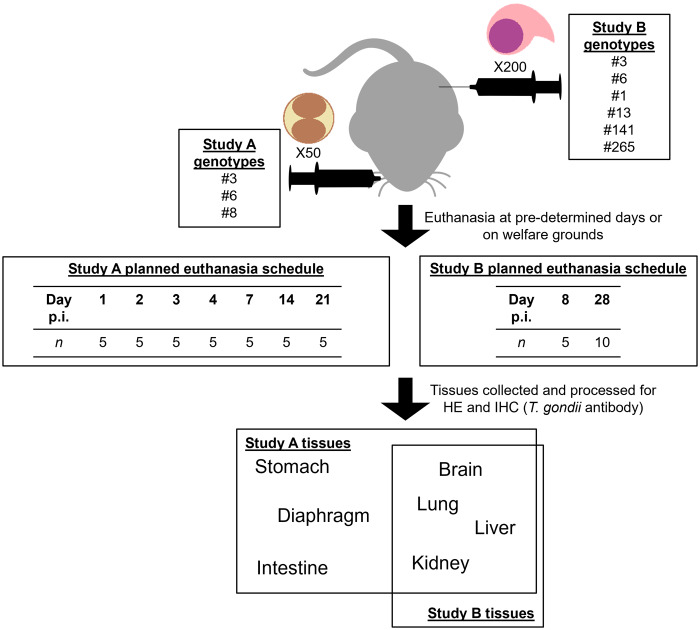
Summary of the workflows for Studies A and B. Figure 1 long description.

For Study B, 120 adult female Swiss CD-1 mice (Hsd:ICR; Envigo RMS, UK) were assigned to 1 of 8 groups (15 mice per group) as shown in Table 2. European genotype #3, and Brazilian genotype #6 described in Study A were used as controls. Mice were intraperitoneally inoculated with 200 *T. gondii* tachyzoites and 5 animals from each group were humanely euthanised at day 8 p.i. The remaining 10 mice from each group were monitored until day 28 p.i. Mice were monitored at least 2 times daily for signs of clinical toxoplasmosis, which were subsequently scored. When signs persisted at the maximum permissible score for 2 consecutive days and recovery was deemed not possible, mice were humanely euthanised (see Hamilton et al. (2019a) for details). Brain and peripheral organs (lung, liver and kidney) were collected at postmortem and fixed in 10% buffered formalin. A graphical summary of the workflows is provided in Figure 1.

**Table 2.** Study B groups and corresponding *T. gondii* isolates Table 2 long description.

Group number	Inoculating isolate	ToxoDB RFLP genotype	Origin of isolate	Reference
B1	TgCkStK12	#1	St. Kitts, Caribbean	Hamilton et al., 2017
B2	TgCkStK13	#141	St. Kitts, Caribbean	Hamilton et al., 2017
B3	TgCkStK10	#141	St. Kitts, Caribbean	Hamilton et al., 2017
B4	TgCkStK2	#265	St. Kitts, Caribbean	Hamilton et al., 2017
B5	TgCkStK9	#13	St. Kitts, Caribbean	Hamilton et al., 2017
B6	TgCkStK11	#13	St. Kitts, Caribbean	Hamilton et al., 2017
B7	Moredun, M4	#3	UK, Europe	Benavides et al. 2011
B8	TgCatBr71	#6	São Paulo, Brazil	Pena et al., 2006

### Histopathology and immunohistochemistry

Formalin-fixed tissues from both studies were routinely processed and embedded in paraffin wax. Blocks were processed for histology (haemotoxylin and eosin [HE]) and immunohistochemistry (IHC) as previously described (Hamilton et al., 2019a; Chiebao et al., 2021). Briefly, sections were cut (5 μm) and stained with HE for histological examination. For IHC, serial sections were cut (5 μm), dried, dewaxed in xylene and taken through alcohols to rehydrate tissues. Slides were then treated with citrate buffer (0.01 M citric acid, pH 6) for heat-induced epitope retrieval. Sections were incubated with normal goat serum (25%) then *T. gondii* rabbit polyclonal antibody (#PA5-16638, Thermo-Fisher Scientific, USA. Immunogen was intact *T. gondii* and used at 1:600) before goat anti-rabbit HRP-labelled polymer (DAKO EnVision+, USA) was applied. AEC (3-amino-9-ethylcarbazole) chromogen solution (Vector Laboratories, UK) was then used to label *T. gondii* red. All tissues were counterstained with haematoxylin and mounted with cover slips. A murine liver infected with *T. gondii* was included as a control, and sections were incubated with *T. gondii* antibody as described above, or with normal rabbit serum (HKV1; 1:500) replacing the *T. gondii* antibody.

### Scoring system for lesions and parasites

Whole sections of brain and peripheral organs were scored blindly using 2 different schemes to account for differences in the pathology observed in different organs. The brain scoring scheme assessed perivascular cuffing (PC), glial foci (GF) and necrotic foci (NF) while the peripheral organ scoring scheme assessed inflammatory infiltrate (II) and NF. In the brain, the number and size of PCs, GF and NF were combined to assess lesions. The frequency of PCs, GF and NF was scored as follows: 0 = absent, 1 = 1–3 foci, 2 = 4–7 foci and 3 = 8 or more foci. The size of PCs, GF and NF was scored based on the proportion of microglia as follows: 0 = not present, 1 = sparse, 2 = moderate and 3 = abundant. The presence of meningitis and any other significant changes were also recorded. In the peripheral organs, the II score was based on the presence of inflammatory cells as follows: 0 = not present, 1 = sparse, 2 = moderate and 3 = abundant. The score for NF was determined by the number found as follows: 0 = absent, 1 = 1–3 foci, 2 = 4–7 foci and 3 = 8 or more foci.

*T. gondii* parasites were labelled and scored in tissues using IHC. A cyst wall–specific antibody was not used in this study meaning it was not possible to determine if tight groups of parasites were cysts containing bradyzoites or parasitophorous vacuoles containing tachyzoites; therefore, tight groups of parasites are referred to as pseudocysts herein. *T. gondii* presence was scored as 0 = absent, 1 = 1–3 pseudocysts or groups of individual tachyzoites, 2 = 4–7 pseudocysts or groups of individual tachyzoites and 3 = 8 or more pseudocysts or groups of individual tachyzoites.

### Statistical analysis

The lesions scored for brain and peripheral organs were different; therefore, the brain and peripheral organs were analysed separately for both studies. The scores for each animal across the measured attributes described above were jointly considered within a multivariate data analysis framework to explore overall patterns and associations according to the groups defined. In particular, principal component analysis (PCA) (Jolliffe and Cadima, 2016) was used to determine optimal combinations of the attributes (principal components; as many as original attributes) providing overall indexes of scores for each animal sample. By construction, the first principle component dimensions account for the highest fraction of total variance in the original data. Thus, the first 2 (denoted Dim1 and Dim2 in graphics) were used to obtain a graphical representation in 2 dimensions known as a biplot. Here, both samples and attributes are respectively represented by points and arrows from the origin, which facilitates a visual assessment of relationships and comparisons at the expense of losing some percentage of information contained in the subsequent principle components. The proximity between the points represents the similarity between samples according to their overall scores across attributes. The arrows indicate directions of increasing scores in the corresponding attributes, with the origin of the biplot (point (0, 0)) representing their average in the original multi-attribute data set. The angle between arrows is proportional to the association, in terms of scores, between the corresponding attributes. As the score data in this work correspond to discrete ordinal variables taking values from 0 to 3, a specialized (nonlinear) variant of PCA was required (unlike ordinary PCA designed for continuous numerical variables). Namely, an optimal scaling procedure (Goodall and Gifi, 1992) was applied, by which the ordinal variables were quantified into a continuous numerical scale making use of step functions in such a way that (1) the variance accounted for in the quantified variables was maximized, and (2) the original order was respected. The resulting transformed (quantified) variables were then used as input in ordinary PCA. Any attribute for which the scores were only zero across all days were not included in the analysis. Moreover, some random noise was added to identical points to facilitate visual distinction in the graphics. Finally, additional information regarding meningitis was included in the brain data sets by projecting its categories (‘Yes’, ‘No’) onto the PCA biplots as supplementary variables. Their coordinates in the graph were determined by the barycentre of the samples belonging to each category.

All data analyses and graphical representations were conducted on the R system for statistical computing v4.4.1 (R Core Team, 2024), using the specialized packages Gifi (Mair et al., 2022) and factoMineR (Lê et al., 2008).

## Results

### Study A: A1 (negative) and A2 (genotype #3) pathology

Peyer’s patches (PPs) of the intestine were developed and active within normal histological limits, in all 5 uninfected mice (A1). No pathological changes or *T. gondii* were observed in group A1 during the study.

Mildly depleted PPs of the intestine were noted in 1 A2 mouse at day 7 p.i. accompanied by mild hepatitis of the liver (semi-suppurative inflammation, eosinophil rich) whilst all other animals had no pathological changes. PPs were active in the majority of A2 mice and *T. gondii* was not identified.

### Study A: A3 (genotype #6) pathology

Group A3 mice (*n* = 5) were euthanised at days 1, 2, 3, 4 and 7 p.i. as planned. Three mice scheduled to be euthanised at day 14 p.i. had to be euthanised at days 8 (*n =* 1) and 10 (*n =* 2) p.i. due to signs of toxoplasmosis (described in Chiebao et al. (2021)). Two mice were euthanised as scheduled on day 14 p.i. as they recovered from signs of toxoplasmosis (Table 3). No samples were collected at day 21 p.i. as all mice had to be euthanised between days 8 (*n =* 1), 9 (*n =* 3) and 11 (*n =* 1) p.i. due to signs of toxoplasmosis. Observations for group A3 are summarized below and in Table 5, but specific scores are presented in Fig. S1.

**Table 3.** Number of animals that reached scheduled euthanasia time points in Study A Table 3 long description.

Group	*Total n*	D1 p.i. (*n*)	D2 p.i. (*n*)	D3 p.i. (*n*)	D4 p.i. (*n*)	D7 p.i. (*n*)	D14 p.i. (*n*)	D21 p.i. (*n*)
A1	5	–	–	–	–	–	–	5/5
A2	35	5/5	5/5	5/5	5/5	5/5	5/5	5/5
A3	35	5/5	5/5	5/5	5/5	5/5	2/5^a^	0/5^b^
A4	35	5/5	5/5	5/5	5/5	5/5	5/5	5/5

a Three A3 animals assigned to day 14 p.i were euthanised prematurely at days 8 (*n =* 1) and 10 (*n =* 2) p.i. on welfare grounds.

b All 5 A3 animals scheduled to be euthanised at day 21 p.i. were euthanised early on welfare grounds at days 8 (*n =* 1), 9 (*n =* 3) and 11 (*n =* 1) p.i.

Pathological changes and *T. gondii* were usually observed from day 7 p.i., although mild intestinal inflammation was present in 1 mouse at day 1 p.i. At day 3 p.i., mild nephritis in the kidney (*n =* 1), inflammation of the diaphragm (*n =* 1), and PP depletion (*n =* 1) were noted in 3 mice. Paneth cell depletion was noted at day 4 p.i. in 1 mouse.

**Figure 2.**
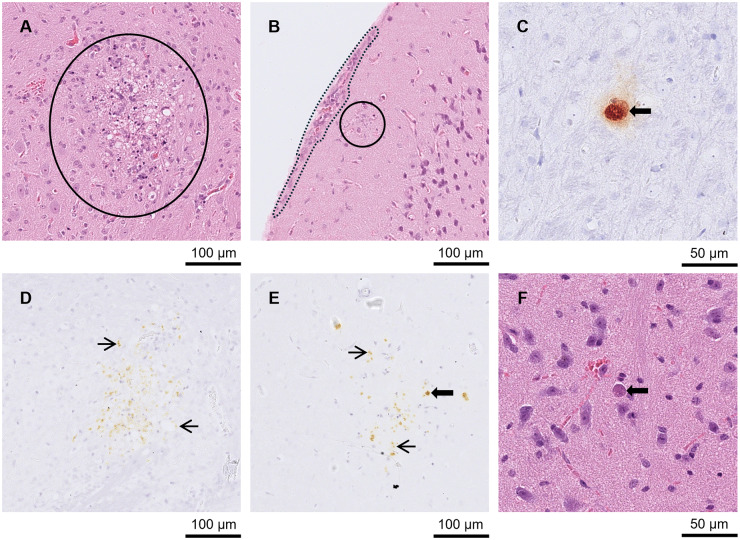
Brain from A3 and A4 mice. (A) and (D) A3 mouse orally inoculated with genotype #6 and euthanised at day 9 p.i. (A) Large area of necrotising inflammation (circled) in HE and (D) tachyzoites labelled by IHC in the same area after serial cutting (arrows). (B) and (E) A3 mouse orally inoculated with genotype #6 and euthanised at day 9 p.i. (B) Moderate meningitis (dotted line) and tachyzoites (solid circle) in HE with the same area (after serial cutting) shown in IHC (E)-Labelled tachyzoites (arrows). (C) and (F) A4 mouse orally inoculated with genotype #8 and euthanised at day 21 p.i. *Toxoplasma gondii* labelled by IHC, (C), was not always directly associated with lesions shown in HE (F). Figure 2 long description.

Lesions were found in the brain from day 9 p.i. Five out of the 6 mice euthanised between days 9 and 11 p.i. had meningitis (Figure 2B and 2E). One of those mice also had large NF (Figure 2A and Figure 2D) and small to moderately sized GF, while 4 other mice had low numbers of small PCs. Between days 8 and 11 p.i. (*n =* 8), all brains had low to high numbers of tachyzoites. Additionally, between days 8 and 11 p.i., 7 of the brains had low to high numbers of pseudocysts. No pathological changes were observed in the brain at day 7 p.i., but 2 mice had low to moderate numbers of tachyzoites. No changes were observed in the 2 brains from mice euthanised at day 14 p.i.

**Figure 3.**
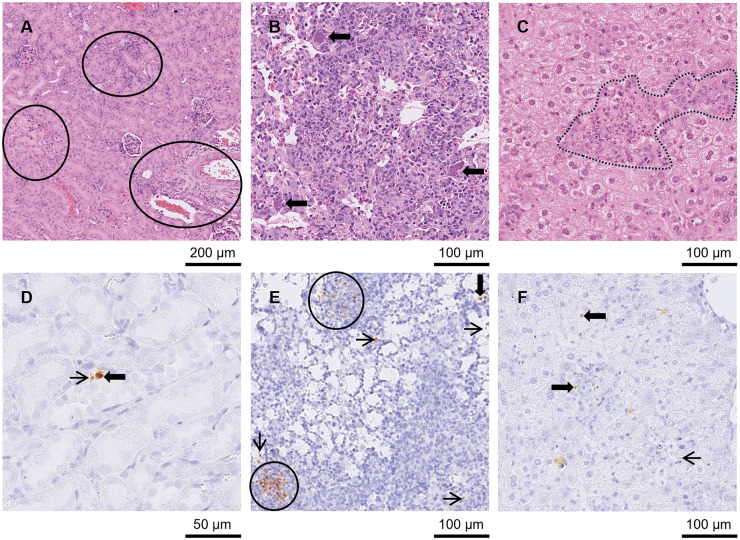
Kidney, lung and liver from groups A3 and A4 mice in Study A. (A) Kidney from an A3 mouse orally inoculated with genotype #6 and euthanised at day 9 p.i. Displaying nephritis and moderate necrotic foci (circled) in HE. (D) Kidney from an A4 mouse orally inoculated with genotype #8 and euthanised at day 14 p.i. With a small pseudocyst (solid arrow) and tachyzoite (thin arrow) in IHC. (B) and (E) Lung from an A4 mouse orally inoculated with genotype #8 and euthanised at day 14 p.i. (B) Necrosis and pneumonia in lung HE surrounded by 3 large pseudocysts (arrows). (E) shows the same area in IHC where pseudocysts (examples given with solid arrows) and groups (outlined in black) or single tachyzoites (examples given with thin arrows) were observed. (C) and (F) Liver from A3 mouse orally inoculated with genotype #6 and euthanised at day 10 p.i. (C) Hepatitis with necrosis and mineralisation in HE (outlined in black) and examples of tachyzoites (thin arrows) and small pseudocysts (solid arrows) in similar area of IHC in (F). Figure 3 long description.

**Figure 4.**
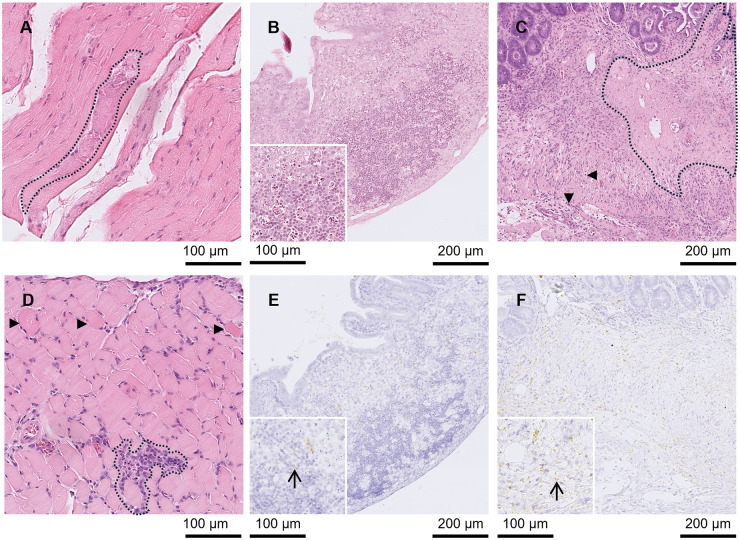
Diaphragm, intestine and stomach from groups A3 and A4 mice. (A) Diaphragm from an A3 mouse orally inoculated with genotype #6 and euthanised at day 10 p.i. Showing mineralisation in HE (outlined in black). (B) and (E) intestinal Peyer’s patches from an A3 mouse orally inoculated with genotype #6 (and euthanised at day 11 p.i. (B) Peyer’s patch induction and depletion in HE and (E) IHC showing various pseudocysts and tachyzoites throughout the same area. Inserts in (B) and (E) show PPs at a higher magnification. A tachyzoite is highlighted by the arrow in (E). Scale bars for the inserts at to the left-hand side of the image. (C) and (F) Stomach from an A3 mouse orally inoculated with genotype #6 and euthanised at day 9 p.i. (C) Gastritis and necrosis in stomach HE. A large area of necrosis was present in the submucosa and the most severely affected area is surrounded by a dotted line. Mononuclear inflammatory cells also infiltrated the submucosa (arrowheads). A large number of tachyzoites were present in IHC, (F), and are highlighted with an arrow in the insert. Scale bars for the inserts at the left-hand side of the image. (D) Diaphragm HE from an A4 mouse orally inoculated with genotype #8 and euthanised at day 14 p.i. Generalized inflammatory cell infiltration with the most severely affected area outlined in black to highlight the presence of inflammatory and necrotic cells. Additionally, several degenerating fibres were present (arrowheads). Figure 4 long description.

Multiple peripheral organs were affected in group A3 mice between days 7 and 11 p.i. During this period, mild to severe hepatitis (*n =* 12) and NF (*n =* 11) were found in the liver. In 1 liver with NF, mineralisation was also present (day 10 p.i.; Figure 3C and 3F). Mild to severe pneumonia (*n =* 10) and moderate to mild NF (*n =* 6) were found in the lung. Pulmonary oedema was reported in 4 mice at days 8 (*n =* 2), 9 (*n =* 1) and 10 (*n =* 1) p.i. and bronchus-associated lymphoid tissue (BALT) induction could be seen in 1 animal at day 9 p.i. Mild to severe nephritis (*n =* 10) and mild NF (*n =* 2) were present in the kidneys (Figure 3A). Low to high numbers of tachyzoites (*n =* 12) and low numbers of pseudocysts (*n =* 11) were also observed. Mild to severe inflammation (*n =* 9), moderate to severe NF (*n =* 4; between day 9 and 11 p.i. only) and mineralisation (*n =* 5; between day 9 and 11 p.i. only) were found in the diaphragm (Figure 4A). Eight diaphragms had low to high numbers of tachyzoites and 6 of these tissues also had low to moderate levels of pseudocysts. Mild to severe inflammation (*n =* 11) and mild to moderate NF were found in the intestine (*n =* 5). Additionally, Paneth cell depletion (*n =* 9) and mild to severe depletion of PPs (*n =* 10) was observed between days 7 and 11 p.i. (Figure 4B and 4E). Tachyzoites were found at low to high numbers (*n =* 12) and pseudocysts were found at moderate to high numbers in the intestine. *T. gondii* was frequently found concentrated at the PPs, villi and lamina propria. Mild to moderate gastritis of the stomach (*n =* 4; only available from 11 mice) and moderate NF (*n =* 1) were present in the stomach (Figure 4C and 4F). Mucosa-associated lymphoid tissue (MALT) was active in a stomach at day 3 p.i. and 2 stomachs at day 9 p.i. In the stomach, tachyzoites ranged from low to high in 6 mice and pseudocysts were mainly low in 5 of these mice. At day 14 p.i., 1 mouse had mild pneumonia in the lung and parasites were absent. In the other mouse, no pathological changes were observed, but a low number of tachyzoites were found in the liver.

### Study A: A4 (genotype #8) pathology

In group A4, 5 mice were euthanised at days 1, 2, 3, 4, 7, 14 and 21 p.i. as planned (Table 3). Observations for group A4 are summarized below and in Table 5, while specific scores are presented in Fig. S2. Pathological changes and *T. gondii* were mainly observed in the peripheral organs from day 7 p.i. and in the brain from day 14 p.i.; however, it was noted that BALT was activated in 3 mice between days 2 and 3 p.i.

At day 7 p.i., the liver was the most affected organ with all mice displaying mild to moderate hepatitis and 1 with multifocal necrosis. Low numbers of tachyzoites were found in 2 livers. Mild intestinal inflammation was observed in 3 mice and PP depletion affected 1 of them. Moderate to high numbers of tachyzoites (*n =* 3) and low to high numbers of pseudocysts (*n =* 4) were also found in some intestines. In the lung, occasional mild pneumonia was observed (*n =* 2) accompanied by BALT induction (*n =* 1) and low numbers of tachyzoites (*n =* 2) and pseudocysts (*n =* 4). The brain, kidney, diaphragm and stomach were unaffected at day 7 p.i.

At day 14 p.i., all brains (*n =* 5) displayed meningitis, low to high numbers of small PCs and low to moderate numbers of small to large GF. In 2 of the mice, the size of GF were variable. In most mice, low to moderate numbers of tachyzoites (*n =* 4) and low to high numbers of pseudocysts (*n =* 5) were present. Severe oedema and pneumonia with moderate to severe necrosis was present in all 5 mice euthanised at day 14 p.i. (Figure 3B) and BALT was induced in 1 case. High numbers of tachyzoites and pseudocysts were found in all lungs examined (Figure 3E). In the liver, mild to severe hepatitis was observed in all animals in addition to mild necrosis in 3 cases, but *T. gondii* was not observed. In the kidney, mild to moderate nephritis occurred in all 5 mice and mild NF was present in 1. One mouse had low numbers of tachyzoites and another had low numbers of pseudocysts (Figure 3D). Mild to severe inflammation of the diaphragm was present in all animals and in 2 animals skeletal muscle degeneration was detected (Figure 4D). A low number of pseudocysts were found in 1 diaphragm. In the intestine mild inflammation was observed in 3 mice and mild depletion of the PP (*n =* 2) or Paneth cells (*n =* 1) was noted. Mild gastritis (*n =* 1) and MALT induction (*n =* 2) was found in the stomach at day 14 p.i. *T. gondii* was not found in the intestine or stomach.

At day 21 p.i., 3 brains had moderate to low numbers of medium to small sized PCs and low numbers of medium to small sized GF. Additionally, 2 were affected by meningitis. Low to high numbers of pseudocysts were found in the 3 brains and low to moderate numbers of tachyzoites were present in 2 (Figure 2C and 2F). The 3 mice with brain pathology also had severe pneumonia with mild to moderate necrosis, oedema and active BALT in the lung. Low numbers of tachyzoites (*n =* 2) and moderate numbers of pseudocysts (*n =* 3) were also identified in the lungs. All mice had mild to moderate hepatitis in the liver and 1 mouse with moderate hepatitis also had mild NF, but no *T. gondii* was found in any case. Mild to moderate nephritis was present in 3 out of the 4 kidneys available for analysis, but *T. gondii* was not observed. Moderate inflammation (*n =* 2) and low numbers of pseudocysts (*n =* 1) were found in the diaphragm. Only 4 diaphragms were available for assessment. Mild PP depletion (*n =* 2) and mild inflammation (*n =* 1) were found in the intestine.

### Study A: Multi-attribute score data analysis for pathology

**Figure 5.**
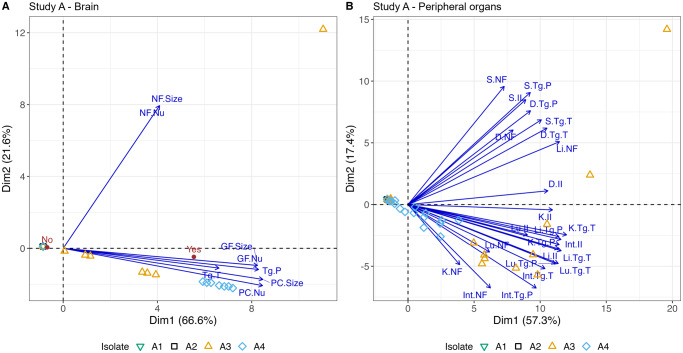
Nonlinear PCA biplots for brain and peripheral organ samples from Study A. Presented in (A) and (B) are the nonlinear PCA biplots for brain (A) and peripheral organs (B) from mice in groups A1, A2, A3 and A4. Scores from all days, lesions and parasite were combined. In the PCA biplot for the brain (A), 2 clear clusters of points were observed, and these corresponded to groups A3 and A4. The distance between the 2 groups suggests the combined scores for A4 were higher than those found in A3 in the brain, and this can be attributed to the increased inflammatory responses (PC, GF) observed in A4 discussed earlier. Data points to the left of the PCA biplot represent zero scores. The arrows show that tachyzoites (Tg.T) had a greater association with A3 while pseudocysts (Tg.P) were associated with both Brazilian isolates but were more abundant in A4. An indication of the average level of meningitis is given by the red points labelled ‘Yes’ and ‘No’. PC.Nu = perivascular cuff (PC) number; PC.Size = size of PC; GF.Nu = glial foci (GF) number; GF.Size = size of GF; NF.Nu = necrotic foci (NF) number; NF.Size = size of NF. In the peripheral organs (B), there were also 2 clear groups that were associated with A3 and A4. Overall scores were higher for A3 than A4 (A3 further to the right of A4). This is likely driven by the often severe levels of pathology and high parasite burdens in A3 mice between days 7 and 11 p.i. Both isolates had a predilection for the lung, liver and intestine of animals and high scores for the stomach, kidney and diaphragm were frequently observed in group A3 and less frequently in A4 as indicated by the arrows. There was a cluster of points to the left of the *y*-axis which are indicative of data points with zero scores and some random noise was added so they were visible. Figure 5 long description.

As most mice in groups A1 and A2 had zero scores, and due to the variability in euthanasia time points for some isolates, it was not feasible to assess scores across *T. gondii* isolates by day; therefore, scores for all time points were combined and assessed by the nonlinear PCA method described above (Figure 5). If a full panel of peripheral organ samples was not available for histopathological analysis, scores for the whole animal were removed from this analysis (*n =* 18). Also, the scoring system used for lesions in the brain (PC, GF, NF) was more complex than that used for peripheral organ analysis as 2 factors, number and size, were included; therefore, this PCA approach was particularly effective for assessing the brain. Upon further investigation (Fig. S3), tachyzoites were found to be the most abundant parasite stage in A3; however, pseudocyst values were not much lower. This analysis also showed that the lung, liver and intestine displayed higher parasite loads than other organs. In A4, pseudocysts appeared more abundant than tachyzoites and they mainly affected the lung, brain and intestine.

### Study B: B1 (genotype #1) pathology

All mice from B1 were euthanised as planned on days 8 (*n =* 5) and 28 (*n =* 10) p.i. (Table 4). A summary of observations for group B1 is given below and in Table 5, while specific scores are presented in Fig. S4.

**Table 4.** Number of animals that reached scheduled euthanasia time points in Study B Table 4 long description.

Group	Total *n*	D8 p.i. (*n*)	D28 p.i. (*n*)
B1	15	5/5	10
B2	15	5/5	5/10^a^
B3	15	5/5	6/10^b^
B4	15	5/5	7/10^c^
B5	15	5/5	0/10^d^
B6	15	5/5	0/10^e^
B7	15	5/5	8/10^f^
B8	15	5/5	0/10^g^

Several animals were euthanised before day 28 p.i. on welfare grounds.

a B2 animals euthanised at days 12 (*n =* 1), 23 (*n =* 2) and 26 (*n =* 2) p.i.

b B3 animals euthanised at days 15 (*n =* 2), 16 (*n =* 1) and 21 (*n =* 1).

c B4 animals euthanised at days 11 (*n =* 2) and 26 (*n =* 1) p.i.

d B5 animals euthanised at days 7 (*n =* 3), 8 (*n =* 6) and 12 (*n =* 1) p.i.

e B6 animals euthanised at days 11 (*n =* 9) and 16 (*n =* 1) p.i.

f B7 animals euthanised at days 16 (*n =* 1) and 21 (*n =* 1).

g B8 animals euthanised at days 8 (*n =* 7), 11 (*n =* 1), 12 (*n =* 1) and 14 (*n =* 1) p.i.

**Table 5.** Summary of organs affected by each *T. gondii* genotype used in this investigation Table 5 long description.

	Study A			Study B		
ToxoDB RFLP genotype	Overall pathogenicity	Organs affected (most → least)	Dominant parasite stage	Overall pathogenicity	Organs affected (most → least)	Dominant parasite stage
#6	Severe	Liver, intestine, lung, kidney, diaphragm, brain, stomach.	Tachyzoites.	Severe	Liver, lung, kidney, brain.	Tachyzoites.
#13	–	–	–	Severe	Lung, liver, kidney, brain.	Tachyzoites.
#8	Moderate	Lung, brain, liver, intestine, diaphragm, kidney, stomach.	Pseudocysts.	–	–	–
#141	–	–	–	Moderate	Brain, lung, liver, kidney.	Brain: pseudocysts; peripheral organs: tachyzoites.
#265	–	–	–	Moderate	Lung, brain, kidney, liver.	Brain: pseudocysts; peripheral organs: tachyzoites.
#3	Mild	Liver, intestine.	Parasite not observed.	Moderate	Brain, lung, liver, kidney.	Brain: pseudocysts; peripheral organ: tachyzoites.
#1	–	–	–	Mild	Brain, lung, liver.	Pseudocysts.

In B1, only moderate to mild hepatitis was observed at day 8 p.i. (*n =* 3) and no *T. gondii* was identified. At day 28 p.i., mild mononuclear meningitis (*n =* 8), moderate to mild numbers of small PCs (*n =* 6) and low numbers of moderately to small sized GF (*n =* 3) were observed in the brain. Moderate to low numbers of *T. gondii* pseudocysts (*n =* 4) and low numbers of tachyzoites (*n =* 2) were discovered in the brain. Mild pneumonia (*n =* 8), mild necrosis (*n =* 1) and BALT activation (*n =* 1) was present in the lung. A low number of pseudocysts were found in 1 lung. Mild hepatitis (*n =* 7) and mild NF (*n =* 1) were observed in the liver. No lesions or parasite were found in B1 kidneys.

### Study B: B2 and B3 (genotype #141) pathology

B2 animals were euthanised at days 8 (*n =* 5) 12 (*n =* 1), 23 (*n =* 2), 26 (*n =* 2) and 28 (*n =* 5) p.i. B3 animals were euthanised at days 8 (*n =* 5) 15 (*n =* 2), 16 (*n =* 1), 21 (*n =* 1) and 28 (*n =* 6) (Table 4). Summaries of observations for groups B2 and B3 are given below and in Table 5, while scores are shown in Figs S5 and S6.

At day 8 p.i. in B2 and B3, no pathology was observed in brains, but a low number of pseudocysts were identified in 1 B2 brain. In B2 peripheral organs at day 8 p.i., mild to moderate pneumonia (*n =* 2) and low numbers of tachyzoites (*n =* 5) and pseudocysts (*n =* 2) were found in the lung. Mild hepatitis (*n =* 2), low numbers of tachyzoites (*n =* 3) and low numbers of pseudocysts (*n =* 2) were found in the liver. It was noted that the hepatic Glisson’s capsule was particularly affected by tachyzoites in 2 cases. Mild nephritis (*n =* 2) and low numbers of tachyzoites (*n =* 1) were found in 2 of the 4 kidneys available for analysis. At day 8 p.i. B3 mice, mild hepatitis (*n =* 2), pneumonia (*n =* 1) and nephritis (*n =* 1) were found in the liver, lung and kidney respectively, but no *T. gondii* was identified.

In the animal from B2 euthanised at day 12 p.i., meningitis, low numbers of small PCs and GFs were observed accompanied by high numbers of *T. gondii* pseudocysts and tachyzoites. Severe pneumonia with mild necrosis and high numbers of pseudocysts and tachyzoites were found in the lung. Moderate hepatitis was found in liver and the kidney was not available for assessment.

**Figure 6.**
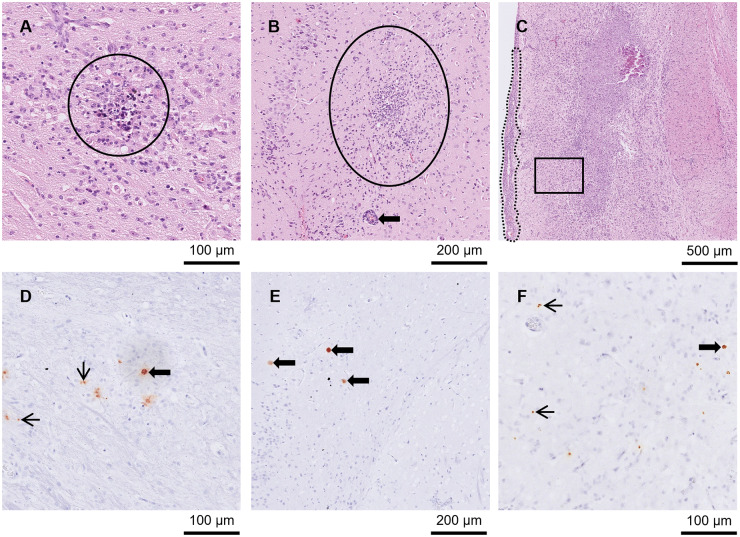
Brain from group B2 and B7 mice. (A) and (D) Brain from a B2 mouse intraperitoneally inoculated with #141 and euthanised at day 23 p.i. (A) Mild necrotizing encephalitis (outlined in black) in HE. (D) IHC-labelled small *T. gondii* pseudocysts (solid arrows) and tachyzoites (groups or singles, thin arrows). (B) and (E) Brain from a B7 mouse intraperitoneally inoculated with genotype #3 and euthanised at day 28 p.i. (B) Moderate glial focus (circled) and mild perivascular cuff (arrow) in HE with IHC-labelled *T. gondii* pseudocysts (arrows) in the same area in (E). (C) and (F) Brain from a B2 mouse intraperitoneally inoculated with #141 and euthanised at day 28 p.i. (C) HE shows a large area of necrosis and severe meningitis (outlined in black) in the brain which was highly populated with *T. gondii* tachyzoites (thin arrows) and pseudocysts (thick arrow) in IHC, (F). Figure 6 long description.

In B2 mice euthanised at days 23 and 26 p.i. (*n =* 4), mononuclear meningitis (*n =* 3) and moderate to high numbers of moderate to large PCs (*n =* 4) that often varied in size (*n =* 3) were observed along with low to high numbers of small (Figure 6A) or large GF (*n =* 4). A large area of necrosis was observed in 1 case (day 23 p.i.) and mineralisation was reported in 3 brains. Low to high numbers of tachyzoites and moderate to high numbers of *T. gondii* pseudocysts were found in all brains (Figure 6D). Mild to severe pneumonia (*n =* 4), pulmonary oedema (*n =* 3), low to moderate numbers of tachyzoites (*n =* 4) and low numbers of pseudocysts (*n =* 2) were reported in lungs. Mild to moderate nephritis (*n =* 2) and low numbers of tachyzoites were observed in kidney (*n =* 1). Mild to moderate hepatitis was noted in 4 livers.

At day 28 p.i., all B2 brains (*n =* 5) showed mononuclear meningitis, mild to moderate numbers of small to large PCs and low to high numbers of small to large GF. One brain also had a low number of large NF (Figure 6C and 6F) and mineralisation was noted in 3 brains. Low to high numbers of pseudocysts were identified in all mice whereas low to high numbers of tachyzoites were present in 3. Mild to severe pneumonia (*n =* 5) with mild necrosis (*n =* 1) or pulmonary oedema (*n =* 1) were identified in addition to low numbers of tachyzoites (*n =* 3) in the lung. Mild hepatitis (*n =* 3) and a low number of pseudocysts (*n =* 1) were found in the liver. Mild nephritis was found in 1 kidney.

B3 mice euthanised at days 15, 16 and 21 p.i. (*n =* 4) had meningitis, low numbers of small PCs and low to moderate numbers of small to moderately sized GF. One brain also displayed low numbers of small NF. Low to high numbers of pseudocysts and tachyzoites were present in all brains.

Pneumonia was severe in all lungs and mild to moderate necrosis (*n =* 2) accompanied by pulmonary oedema (*n =* 1) was observed in addition to low numbers of tachyzoites (*n =* 3) and pseudocysts (*n =* 2). Mild to moderate hepatitis (*n =* 3) and low numbers of pseudocysts were found in the liver. Mild nephritis was also observed in 2 kidneys.

At day 28 p.i. in B3 (*n =* 6), meningitis (*n =* 6), low to high numbers of small (*n =* 1) or large (*n =* 4) PCs and low to high numbers of small (*n =* 1), moderate (*n =* 3) or large (*n =* 1) GF were present in the brain. Low numbers of tachyzoites (*n =* 4) and low to high numbers of pseudocysts (*n =* 6) were also found in the brains. Moderate to severe pneumonia affected all mice and at times was associated with mild to moderate necrosis (*n =* 5), pulmonary oedema (*n =* 3) and BALT induction (*n =* 2). Low numbers of tachyzoites were present in 2 lungs. Mild to moderate hepatitis (*n =* 6) and low numbers of tachyzoites were found in the liver. Mild to moderate nephritis (*n =* 4) was observed in the kidney.

### Study B: B4 (genotype #265) pathology

B4 animals were euthanised at days 8 (*n =* 5), 11 (*n =* 2), 26 (*n =* 1) and 28 (*n =* 7) p.i. (Table 4). A summary of observations for group B4 is given below and in Table 5, while specific scores are shown in Fig. S7. Mice euthanised at day 8 p.i. (*n =* 5) displayed pathology in the lung and liver. Mild pneumonia (*n =* 2), mild pulmonary oedema (*n =* 1) and low numbers of tachyzoites (n = 2) were present in the lung. Mild hepatitis (*n =* 1), low numbers of tachyzoites and 1 pseudocyst were identified in the liver. Lesions were not present in the brain or kidney, but low numbers of tachyzoites were present in both (brain *n* = 1; kidney *n* = 2).

**Figure 7.**
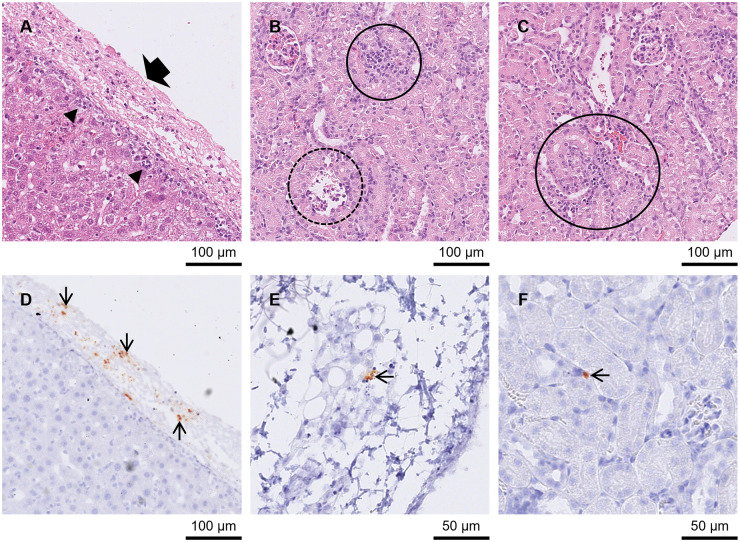
Liver and kidney from groups B4–B6 mice. (A) and (D) Liver from a B5 mouse intraperitoneally inoculated with #13 and euthanised at day 8 p.i. (A) Moderate hepatitis at edge of liver HE section (arrowheads) with inflammation of the Glisson’s capsule (large arrows) which had a high *T. gondii* tachyzoite presence in IHC (arrows), (D). (B) and (E) Kidney from a B4 mouse intraperitoneally inoculated with #265 and euthanised at day 11 p.i. (B) Moderate necrotizing nephritis (solid circle) with inflammatory infiltrate (dotted circle) in HE. (E) Small cluster of IHC labelled *T. gondii* tachyzoites (arrow) in renal capsule. (C) and (F) Kidneys from 2 B6 mice intraperitoneally inoculated with #13 and euthanised at day 11 p.i. **(C)** necrotizing nephritis (circled) in the kidney HE. (F) Tachyzoite (arrow) IHC labelled in the main body of the kidney. Figure 7 long description.

At day 11 p.i. (*n =* 2), both mice presented with meningitis, with 1 also showing low numbers of small PCs and GF. High numbers of pseudocysts and low to moderate numbers of tachyzoites were identified in both brains. Moderate and severe pneumonia affected both lungs and 1 also had mild necrosis. Moderate or high numbers of tachyzoites and low and moderate numbers of pseudocysts were found in each lung. Moderate and severe nephritis and low or moderate numbers of tachyzoites were observed in the kidney (Figure 7B). It was noted that the renal capsule was concentrated with *T. gondii* (Figure 7E). Moderate hepatitis was present in both mice, and 1 also had mild necrosis. Low or moderate numbers of tachyzoites (*n =* 2) low numbers of pseudocysts (*n =* 1) were also identified in the liver. *Toxoplasma gondii* was abundant in the Glisson’s capsule.

At day 26 and 28 p.i. (*n =* 8), all brains displayed meningitis, low to high numbers of small to large PCs and low to moderate numbers of small to large GF. Pseudocysts were present in the brain at low to moderate numbers (*n =* 7), and few tachyzoites were present (*n =* 5). Mild to severe pneumonia (*n =* 6), pulmonary oedema (*n =* 1), BALT induction (*n =* 2) and low numbers of *T. gondii* pseudocysts (*n =* 2) and tachyzoites (*n =* 1) were observed in the lung. Mild hepatitis (*n =* 4) and nephritis (*n =* 2) were observed in the liver and kidney, respectively.

### Study B: B5 and B6 (genotype #13) pathology

B5 animals were euthanised at days 7 (*n =* 3), 8 (*n =* 11) and 12 (*n =* 1) p.i. B6 animals were euthanised at days 8 (*n =* 5), 11 (*n =* 9) and 16 (*n =* 1) p.i. (Table 4). A summary of observations for groups B5 and B6 are presented below and in Table 5, while specific scores are shown in Figs S8 and S9.

**Figure 8.**
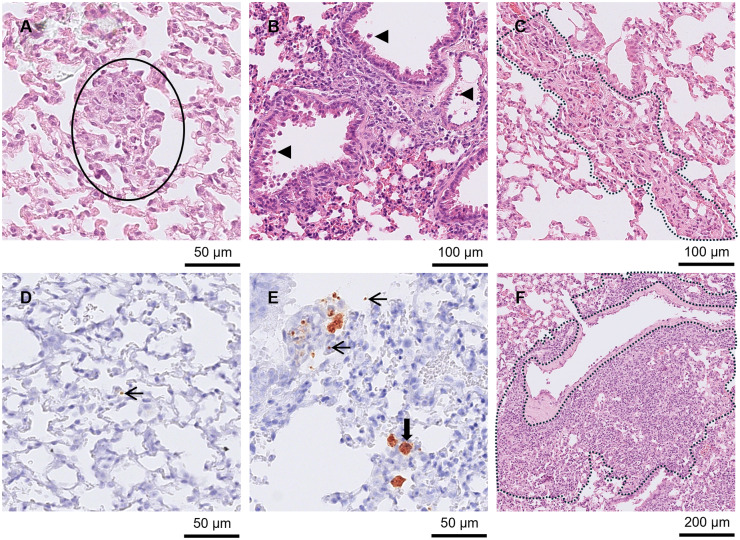
Lung from group B5 and B7 mice. (A), (C) and (D) Lung from a B7 mouse intraperitoneally inoculated with genotype #3 and euthanised at day 8 p.i. (A) Small necrotic foci in HE (outlined in black) with tachyzoite (arrow) labelled in the same area in IHC, (D). Also present in the HE section was vasculitis (outlined in black), (C). (B) and (E) Lung from a B5 mouse intraperitoneally inoculated with #13 and euthanised at day 8 p.i. (B) Moderate interstitial necrotizing pneumonia in HE with pseudocysts (solid arrow) and tachyzoites (thin arrow) labelled in the same tissue by IHC, (E). Additionally, there were inflammatory cells (arrowheads) in the bronchi in B). (F) Lung from a B7 mouse intraperitoneally inoculated with genotype #3 and euthanised at day 21 p.i. with induced and depleted BALT in HE (outlined in black). Figure 8 long description.

In B5 at days 7 and 8 p.i., low numbers of tachyzoites (*n =* 4) and pseudocysts (*n =* 1) were occasionally found in the brain, but no pathological changes were identified. At day 7 p.i. mild hepatitis (*n =* 3) with mild necrosis (*n =* 2), low to high numbers of tachyzoites (*n =* 3) and low to moderate numbers of pseudocysts (*n =* 2) were present in the liver. It was noted that *T. gondii* was often concentrated in the Glisson’s capsule. Low to high numbers of tachyzoites (*n =* 2), low to moderate numbers of pseudocysts (*n =* 1) and mild nephritis (*n* = 1) were found in the kidney. The lung had low to high numbers of tachyzoites (*n =* 3) and high numbers of pseudocysts (*n =* 1) but no lesions. At day 8 p.i., there was moderate to severe hepatitis (*n =* 10) with mild necrosis (*n =* 2) and low to high numbers of tachyzoites (*n =* 10) and pseudocysts (*n =* 8) in the liver. Inflammation and *T. gondii* were concentrated at the periphery of the tissue and Glisson’s capsule of 4 livers (Figure 7A and 7D). Moderate to severe pneumonia (*n =* 7) with mild necrosis (*n =* 3), pulmonary oedema (*n =* 1) and BALT induction (*n =* 2) were identified in the lung along with low to high numbers of tachyzoites (*n =* 11) and pseudocysts (*n =* 8; Figure 8B and 8E). Mild or moderate nephritis was present in 6 animals. Low to moderate numbers of tachyzoites (*n =* 7) and low numbers of pseudocysts (*n =* 3) were found in the kidney.

At day 12 p.i. (*n =* 1) for B5, low numbers of small PCs and GF were present in the brain. High numbers of tachyzoites and low numbers of pseudocysts were also found. Severe pneumonia and high numbers of pseudocysts and tachyzoites were found in the lung. Moderate hepatitis and low numbers of pseudocysts and tachyzoites were observed in the liver. Low numbers of tachyzoites were found in the kidney, but no lesions were identified.

At day 8 p.i. (*n =* 5) in B6, mild to moderate pneumonia (*n =* 3), low to high numbers of tachyzoites (*n* = 5) and low to moderate numbers of pseudocysts (*n* = 3) were present in the lung. Mild to moderate hepatitis (*n =* 3) and low numbers of tachyzoites (*n =* 2) and pseudocysts (*n* = 1) were found in the liver. Mild nephritis (*n =* 2) and low numbers of tachyzoites (*n* = 1) were found in the kidney. Low numbers of tachyzoites were found in 2 brains and no pathological changes were noted.

Mild neuropathology was observed in the brain from day 11 p.i. (*n =* 9) in B6. Mice presented with meningitis (*n =* 5), low numbers of small PCs (*n =* 3) and GF (*n =* 3) in the brain. Moderate to high numbers of tachyzoites were identified in all brains (*n =* 9) and low to moderate numbers of pseudocysts were found in most brains (*n =* 7). Moderate to severe pneumonia (*n =* 8) and mild to moderate necrosis (*n =* 6) could be seen in the lung. It was noted that in the lung without pneumonia, there was mild necrosis. Tachyzoites and pseudocysts were predominantly high in number in the lungs (*n =* 8 and *n =* 7, respectively) but moderate in a few cases (*n =* 1 and *n =* 2, respectively). Moderate to severe hepatitis (*n =* 7) was abundant in the liver but mild hepatitis was seen in 1 case. Mild necrosis (*n =* 2) was occasionally observed, including in 1 liver with no hepatitis. Low to moderate numbers of tachyzoites (*n =* 8) and pseudocysts (*n =* 6) were identified. Moderate to severe nephritis (*n =* 7) was observed in most kidneys (Figure 7C), but mild nephritis was found in 1 case. Low to moderate numbers of tachyzoites and a low number of pseudocysts were found in some kidneys (*n =* 6 and *n =* 1 respectively; Figure 7F).

At day 16 p.i. (*n =* 1), meningitis and a moderate number of small PCs and GF were found in the brain along with high numbers of pseudocysts and tachyzoites. Additionally, a large area of necrotising inflammation was identified in the brain. Severe pneumonia with mild necrosis and BALT activation was also observed in the lung. There was also moderate hepatitis in the liver and *T. gondii* was found in low numbers in the form of tachyzoites.

### Study B: B7 (genotype #3) pathology

Group B7 animals were euthanised at days 8 (*n =* 5), 16 (*n =* 1), 21 (*n =* 1) and 28 (*n =* 8) p.i. (Table 4). A summary of observations for group B7 is given below and in Table 5, while specific scores are shown in Fig. S10.

Lesions were not observed in the brain at day 8 p.i. in B7, but pseudocysts were found at low numbers in 1 brain. Mild to moderate pneumonia (*n =* 3), occasionally with mild necrosis (*n =* 1) and low numbers of tachyzoites (*n =* 3) and pseudocysts (*n =* 1) were identified in the lung (Figure 8A, 8C and 8D). Mild hepatitis (*n =* 3) and mild necrosis (*n =* 1) were observed in the liver in addition to low numbers of tachyzoites (*n =* 1) and pseudocysts (*n =* 2). Mild nephritis (*n =* 1) was found in the kidney along with low numbers of tachyzoites (*n =* 1) and pseudocysts (*n =* 1).

The brain from the mouse euthanised at day 16 p.i. had meningitis, low numbers of small PCs, moderate numbers of small GF, moderate numbers of tachyzoites and high numbers of pseudocysts. There was also severe pneumonia and pulmonary oedema in the lung accompanied by high numbers of pseudocysts and tachyzoites. Moderate nephritis and low numbers of tachyzoites were present in the kidney. In the liver, mild hepatitis and low numbers of tachyzoites were found.

At day 21 p.i. (*n =* 1), the brain showed severe meningitis, a high number of moderately sized PCs and a moderate number of variably sized GF which could be large and associated with mineralisation. There were also a high number of pseudocysts and a low number of tachyzoites. Severe pneumonia, BALT activation and depletion and moderate numbers of tachyzoites and low numbers of pseudocysts were seen in the lung (Figure 8F). Moderate nephritis of the kidney and moderate hepatitis of the liver were also observed.

At day 28 p.i. (*n =* 8), all animals had mononuclear meningitis and in 3 of these animals, most cells resembled lymphocytes. Low to high numbers of small to large PCs were also found in all brains. Seven also had low to high numbers of small to large GF and in 4 cases, GF varied in size within the brain (Figure 6B and 6E). Low to high numbers of pseudocysts (*n =* 7) and low numbers of tachyzoites (*n =* 5) were present in some cases. Mild to severe pneumonia (*n =* 8) occasionally accompanied by mild necrosis (*n =* 1), BALT induction (*n =* 2) or oedema (*n =* 1) and low numbers of tachyzoites (*n* = 3) were present in lung. Mild hepatitis (*n =* 2) and low numbers of tachyzoites (*n* = 1) were present in the liver. No pathological changes or *T. gondii* were found in the kidney.

### Study B: B8 (genotype #6) pathology

Group B8 animals were euthanised at days 8 (*n =* 12), 11 (*n =* 1), 12 (*n =* 1) and 14 (*n =* 1) p.i. (Table 4). A summary of observations for group B8 is given below and in Table 5, while scores are shown in Fig. S11. In group B8, brain lesions were present in 1 animal at day 8 p.i. in the form of a low number of small GF. Low numbers of tachyzoites and pseudocysts were found in another brain at day 8 p.i. At days 11, 12 and 14 p.i., moderate to high numbers of tachyzoites were found in all brains (*n* = 3), while low to moderate numbers of pseudocysts were found in 2.

Peripheral organs from mice euthanised at days 8 and 11 p.i. displayed similar pathology; therefore, they are described together. In the peripheral organs at days 8 and 11 p.i. (*n =* 13), mild to moderate pneumonia (*n =* 8), low to high numbers of tachyzoites (*n* = 13) and pseudocysts (*n =* 10) were present in lungs.

Mild to moderate hepatitis (*n =* 10) and low to high numbers of tachyzoites (*n* = 13) and pseudocysts (*n =* 8) were present in livers. It was noted that the Glisson’s capsule of most livers (*n =* 9) had a high proportion of labelled *T. gondii*. Only 12 kidney samples were available for histopathological assessment at days 8 and 11 p.i. In the kidney, mild nephritis (*n =* 1), low to moderate numbers of pseudocysts (*n =* 4) and low numbers of tachyzoites (*n =* 9) were present. The renal capsule of kidneys (*n =* 5) had a high proportion of labelled *T. gondii*.

At day 12 and 14 p.i. (*n =* 2), pathology was similar; therefore, the 2 animals are described together. Moderate to severe hepatitis with mild to moderate necrosis, high numbers of tachyzoites and pseudocysts were found in the livers. The Glisson’s capsule of 1 liver was particularly positive for *T. gondii*. In the lungs, there was mild or severe pneumonia with mild to moderate necrosis and high numbers of tachyzoites and pseudocysts. Mild nephritis and high numbers of tachyzoites were identified in both kidneys. One kidney also had low numbers of pseudocysts.

### Study B: Multi-attribute score data analysis for pathology

**Figure 9.**
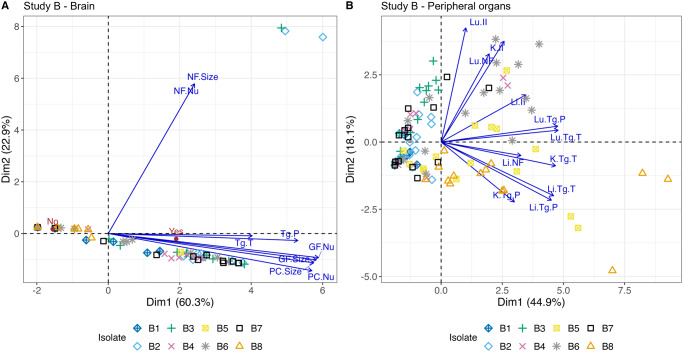
Nonlinear PCA biplot for brain and peripheral organ samples from Study B. Presented in (A) and (B) are the nonlinear PCA biplots for brain (A) and peripheral organs (B) from mice in groups B1 through to B8. Scores from all days, lesions and parasite were combined. In the brain (A), scores for B2, B3, B4, B6 and B7 were concentrated on the right of 0 on the *x*-axis indicating there were higher overall pathology and *T. gondii* presence compared to B5 and B8, where most points were located to the left. B1 were around zero on each side of the *x*-axis, consistent with mild to moderate pathology and parasite presence in the brain. Three outliers on the right belonging to B2 and B3 reflect the presence of NF. In the peripheral organs (B), many isolates that dominated the right-hand side of the graph in the brain were predominantly found on the left-hand side of the peripheral organ PCA biplot (B2, B3, B4 and B7). The opposite trend also appeared; those isolates that dominated the left-hand side of the brain PCA biplot were found more often on the right-hand side of the peripheral organ graph (B5, B6 and B8). B1 continued to be associated with low lesion and parasite scores in the peripheral organs and was positioned on the left of the *x*-axis. Figure 9 long description.

Further assessment using combined scores and nonlinear PCA biplots were conducted for Study B samples (Figure 9). If a full panel of peripheral organ samples was not available for histopathological analysis, scores for the whole animal were removed from peripheral organ analysis (*n =* 4).

Loosely, the nonlinear PCA panels in Figure 9 can be associated with organs, for example, points concentrated above 0 on the *y*-axis were often associated with higher overall lung scores, while points below 0 on the *y*-axis were often associated with higher scores in the liver. Additionally, points located on the left of 0 on the *x*-axis often had low to moderate scores while those found on the right of the *x*-axis often had moderate to severe scores. B1 was associated with low scores and points were found on either side of the *y*-axis close to 0. The points below 0 likely reflect the higher scores found in livers at day 8 p.i., while those slightly above 0 likely reflect an addition of scores in the lung at day 28 p.i. B2 and B3 scores were found on the left of the *x*-axis indicating mild to moderate scores. B2 scores were distributed above and below 0 of the *y*-axis as a result of mild to moderate scores in the liver and lung, while B3 appeared to dominantly affect the lungs although scores were mild to moderate in the liver on some occasions. B4 had higher scores in the lung, reflected in the presence of some points above the *y*-axis. Two points were found on the right of the *x*-axis and were likely driven by the presence of higher kidney scores in 2 animals. B5 affected the lung and liver most with some scores in the kidney, explaining the presence of data points in 3 panels of the plot. In this isolate, the scores were often higher because of parasite burden more than lesion severity. Most scores for B6 were concentrated in the upper left of the plot, probably because of high parasite and lesions scores found in the lung. The liver and kidney were often affected by moderate to severe pathology, but parasite scores were not as high potentially explaining the presence of some points to the left of the *x*-axis. Some animals in B7 had high scores in the lung, others had moderate scores in the lung and/or liver and/or kidney, while the rest had low or absent scores across the peripheral organs. This explains the range of points throughout 3 of the panels. B8 caused high overall scores in the lung and liver, but scores were consistently present in the kidney too, usually driven by high parasite loads. Scores were also often high in the other organs because of high parasite burdens. The addition of consistent scores in the kidney likely explains why data points for B8 were concentrated below 0 on the *y*-axis.

Further analysis of *T. gondii* life stage was assessed in Fig. S12. Pseudocysts were the dominant *T. gondii* life stage found in all B1 organs and B2, B3, B4 and B7 brains. Tachyzoites were the main life stage found in all tissues infected with B5, B6 and B8 and dominated the peripheral organs in B2, B3, B4 and B7.

### Comparison of pathology caused by different parasite life stage

**Figure 10.**
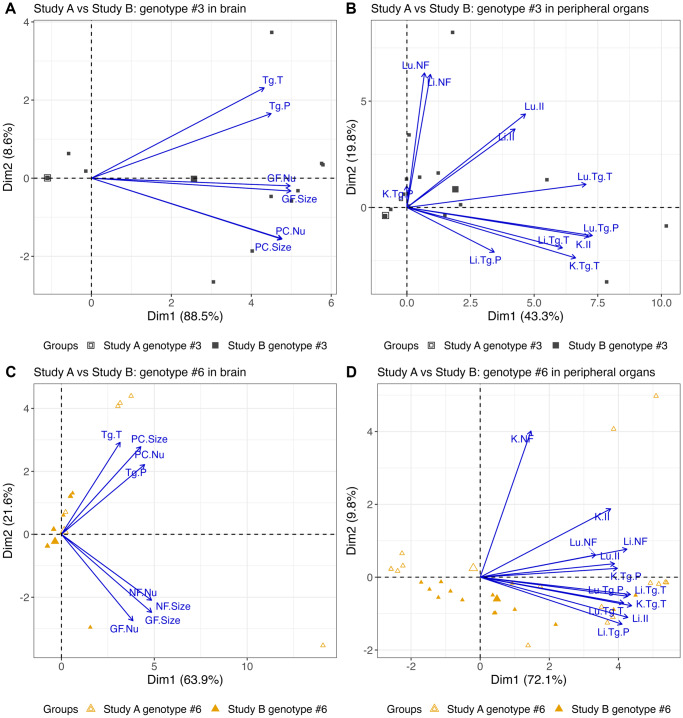
Nonlinear PCA biplots for brain and peripheral organs comparing scores for genotypes #3 and #6 in both studies. Genotypes #3 and #6 were used in both Studies A and B, but mice were orally inoculated with 50 oocysts in Study A and intraperitoneally inoculated with 200 tachyzoites in Study B. Additionally, the days at which the mice were euthanised at varied between studies and more organs were scored in Study A compared to Study B. Combining the scores allows preliminary assessment of genotypes #3 and #6 in 2 life stages. In the brain (A) and peripheral organs (B), genotype #3 from Study A, inoculated by ingestion of oocysts, produced zero or low scores while genotype #3 from Study B, inoculated by intraperitoneal injection of tachyzoites, caused higher scores for pathology and parasite burden in all organs. For genotype #6, in the brain (C), overall scores were higher in Study A than in Study B as it was more likely to see lesions and parasite. Parasite burdens, when present, appeared to be similar. In the peripheral organs present in both studies (lung, liver and kidney), Studies A and B showed moderate to severe lesion and parasite scores (D). Figure 10 long description.

Nonlinear PCA biplots containing the brain or peripheral organ (lung, liver and kidney only) scores for genotype #3 from Studies A and B show there were differences in scores between the studies (Figure 10).

The apparent grouping that occurred in the PCA biplot may be explained by the difference in the days mice were euthanised on between the studies. For example, between days 8 and 12 p.i., scores were high in both studies and may explain the mix of data points towards the right of the *x*-axis, whereas the groups consisting primarily of Study A or Study B data points may relate to days out with this period where scores were lower.

## Discussion

In this study, canonical and non-canonical strains of *T. gondii* showed visible differences in the severity of pathology observed in murine tissues, where genotype #6 and #13 isolates were most pathogenic, genotypes #8, #141 and #265 were moderately pathogenic and genotype #1 showed little pathogenicity. The pathogenicity for genotype #3 depended on route of infection, where an oral infection with oocysts resulted in mild pathogenicity and intraperitoneal infection with tachyzoites caused moderate pathogenicity.

The order in which organs were affected by *T. gondii* in both studies reflected the expected dissemination route the parasite would take from the respective inoculation sites. For those orally inoculated, *T. gondii* likely moved from the intestine to lymphatic and circulatory systems, through the portal blood stream to the liver, then into the systemic circulatory system (Denk et al., 2022). Once in the bloodstream, it could be surmised that *T. gondii* tachyzoites left the circulation and entered organs in order of first contact beginning in the liver, then to the kidney, lung (Ajzenberg et al., 2009; Unno et al., 2013; Jurankova et al., 2015) and lastly the brain (Jurankova et al., 2015).

In A3 mice inoculated with the genotype #6 isolate, damage in the intestine can be explained by the presence of excysted sporozoites infecting intestinal cells which subsequently rapidly proliferated. PP depletion indicates an active infection (Liesenfeld et al., 1997) and has previously been reported in association with severe intestinal pathology after oral ingestion of *T. gondii* (Suzuki et al., 2000) and lethal toxoplasmosis (Mordue et al., 2001). Additional Paneth cell depletion observed can result in changes to the intestinal microbiota further consolidating intestinal inflammation and pathology (Raetz et al., 2013).

Previously, enteric lesions associated with severe enteritis and mesenteric lymphadenitis were thought to cause early mortality in orally inoculated mice, while mice that survived succumbed to myocarditis, pneumonia and encephalitis (Dubey and Frenkel, 1973). While the previous reports account for some of the clinical and pathological findings in A3, systemic toxoplasmosis characterized by parasitaemia and overstimulation of the immune response, in addition to type of immune response, may also have contributed to morbidity and mortality (Chiebao et al., 2021; Denk et al., 2022). This is proposed as, in most brains from A3 mice euthanised before their scheduled endpoints, lesions did not correspond to the severity of the parasite numbers suggesting genotype #6 was able to replicate substantially before a glial response could occur. Further, the high number of tachyzoites and severity of pathology throughout A3 tissues strongly suggest pseudocysts represented parasitophorous vacuoles, within which tachyzoites underwent rapid replication before lysis (Rodrigues Oliveira et al., 2022); however, further assessment is required to confirm the absence of a cyst wall.

In the 2 A3 mice that recovered by day 14 p.i, the immune response may have been adequate to control infection; however, methodological variations need to be considered: variation in viable oocyst numbers per inocula due to the inhomogeneous nature of an inoculating suspension; variation in oocyst excystation rate and infection rate of intestinal cells; and, given the use of outbred mice, genetic variability can allow mice to respond differently to infections.

A key difference in pathology between groups A3 and A4 was the apparent resolution of earlier liver and intestinal pathology as organs supplied later in the circularity system, including the brain and lungs, became more affected by genotype #8 *T. gondii*. This difference may be explained by *T. gondii* morphology; pseudocysts were the dominant life stage identified suggesting genotype #8 tachyzoites replicated slowly in parasitophorous vacuoles or they differentiated into bradyzoites in cysts, indicating the development of a chronic infection (Remington et al., 2006). Meanwhile, genotype #6 sustained severe pathology throughout tissues.

In intraperitoneally inoculated mice, the origin of infection was reflected in the early pathology found in abdominal organs assessed; however, the subsequent spread of infection throughout organs was similar to those orally inoculated. For the highly pathogenic genotype #13 and #6 isolates inoculated into groups B5 and B8, respectively, the hepatic Glisson’s capsule was positive for *T. gondii* early in infection (days 8 and 12 p.i.) suggesting infiltration of tachyzoites from the peritoneal inoculation site to the liver. Additionally, pathology and parasite presence in the kidney was higher in groups inoculated with highly pathogenic isolates compared with other isolates investigated in this study.

For the moderately pathogenic *T. gondii* isolates, there were minor variations in the order in which organs were affected (Table 5), and mice presented with pseudocysts predominantly in the brain and tachyzoites predominantly in the peripheral organs. This contrasted with the pseudocyst dominant infection found throughout organs in the moderately pathogenic isolate in Study A suggesting that the inoculation method also influences pathology in moderately pathogenic *T. gondii* isolates. The continued observation of rapidly dividing tachyzoites in the periphery could account for the differences in mortality rates between moderately pathogenic isolates in Studies A and B.

Further evidence of the importance of inoculation route is highlighted by the change in genotype #3 pathogenicity from mild to moderate in Studies A and B, respectively. There are several reasons for increased pathogenicity when tachyzoites are intraperitoneally inoculated including: *T. gondii* bypassed gastric digestion and the intestinal barrier that could have prevented parasitic infection if inoculated orally; tachyzoites are more infectious or infectious for longer than sporozoites released from oocysts (Dubey and Frenkel, 1973, 1976); different breeds of outbred mice were used in the studies and the methodological variations discussed previously for oocysts must be considered.

Genotype #1 used to inoculate group B1 mice showed very mild pathogenicity. The low numbers of *T. gondii* observed in tissues suggests this isolate may not disseminate as much as more pathogenic strains (Gavrilescu and Denkers, 2001) and the presence of mild inflammatory lesions indicate the host immune response was adequate to control infection and limit secondary complications.

Genotype #3 and #6 isolates were used in Studies A and B, but the method of inoculation was different allowing a comparison of pathology induced by the different methods. The main limitations of comparing the results from the 2 studies were varying euthanasia schedules; differences in parasite stages and doses; different inoculation methods; and different organs examined. Each of the limitations were considered in turn when determining the conclusions.

To account for the differences in tissue availability, only tissues shared in both studies were compared in the analysis of genotypes #3 and #6. Using PCA as the statistical method allowed all pathology and parasite scores to be compared between isolates independent of euthanasia schedules. For genotype #6, animals in both studies were euthanised no later than day 14 p.i.; therefore, euthanasia schedules were not likely to affect assessment. For genotype #3, mice euthanised between day 8 and 28 p.i. in Study B had consistently higher scores than those in Study A at similar time points; therefore, it could be concluded that the differences in pathology were due to something other than euthanasia schedules, for example inoculation route, as described earlier, or the inoculant dose. A sporulated oocyst contains 2 sporocysts, which house 4 sporozoites each (Dubey, 1998). Inoculating 50 oocysts orally is the equivalent of 400 sporozoites being ingested; therefore, potentially 400 parasites able to infect intestinal cells, differentiate into tachyzoites, multiply and enter the circulation. Therefore, theoretically, the animals in Study A were inoculated with more *T. gondii* than animals in Study B. Despite this, at least for genotype #3, the mice inoculated with 200 tachyzoites had higher pathology and parasite scores in the brain and peripheral organs compared to those who received the higher infectious dose of oocysts, corroborating findings from an early paper (Dubey and Frenkel, 1973). For genotype #6, the differences between oocyst and tachyzoite infection were less obvious; however, it is known that highly virulent strains, like those in genotype #6, reach high parasite loads regardless of inoculation dose due to shorter doubling times, resistance to host immune system, reduced conversion to the bradyzoite stage and/or higher reinvasion rate (Saeij et al., 2005).

Regardless of the limitations, having formalin-fixed parafin-embedded material from 2 studies investigating multiple *T. gondii* isolates provided the unique opportunity to interrogate pathological changes caused by *T. gondii*. This study showed less pathogenic strains can establish infection in the brain in the form of pseudocysts/cysts and highly pathogenic strains can multiply so quickly they become systemic and cause widespread damage to tissues, parasitaemia and overstimulation of the immune response leading to death. Additionally, the route of inoculation/infective stage can influence the pathology caused by less virulent strains but has no apparent effect on the pathology caused by highly virulent strains. The results from the current study will improve the general understanding of the pathological changes induced by non-canonical strains and will help to refine the management and treatment of toxoplasmosis.

## Supporting information

10.1017/S0031182026101589.sm001 Black et al. supplementary material 1 Black et al. supplementary material

10.1017/S0031182026101589.sm002 Black et al. supplementary material 2 Black et al. supplementary material

## Supplementary material

The supplementary material for this article can be found at http://doi.org/10.1017/S0031182026101589. Additional data can be made available upon request to the corresponding author.

## Table 1 Long description

The table maps Study A group numbers (A1–A4) to the inoculating T. gondii isolate, its ToxoDB RFLP genotype, origin, and literature reference. Group A1 contains only dashes, indicating no isolate, genotype, origin, or reference is provided. Group A2 uses isolate Moredun M4 with genotype #3 from the UK (Europe), cited to Benavides et al., 2011. Groups A3 and A4 use Brazilian isolates from São Paulo: TgCatBr71 (genotype #6) and TgCatBr60 (genotype #8), both cited to Pena et al., 2006. The only repeated origin is São Paulo, Brazil (two groups), while the UK appears once. No outcomes or sample sizes are included, so the table supports identification of isolates and sources rather than comparisons of experimental results.

Navigate back to Table 1.

## Table 2 Long description

The table links Study B group numbers (B1–B8) to the inoculating Toxoplasma gondii isolate, its ToxoDB RFLP genotype, geographic origin, and literature reference. Six groups (B1–B6) use isolates from St. Kitts in the Caribbean: TgCkStK12 genotype #1; TgCkStK13 and TgCkStK10 both genotype #141; TgCkStK2 genotype #265; and TgCkStK9 and TgCkStK11 both genotype #13. Two groups use non–St. Kitts isolates: Moredun, M4 genotype #3 from the UK (Europe) and TgCatBr71 genotype #6 from São Paulo, Brazil. Repeated genotypes occur within St. Kitts groups (#141 appears in B2 and B3; #13 appears in B5 and B6), while other genotypes are unique to a single group. References are Hamilton et al., 2017 for all St. Kitts isolates, Benavides et al., 2011 for the UK isolate, and Pena et al., 2006 for the Brazil isolate. The table provides identifiers and origins but does not include outcomes or sample sizes, so no conclusions about virulence or study results can be drawn from it alone.

Navigate back to Table 2.

## Figure 1 Long description

The diagram illustrates workflows for Studies A and B. Study A involves 50 genotypes (#3, #6, #8), while Study B involves 200 genotypes (#6, #1, #13, #141, #265). Euthanasia occurs at predetermined days or on welfare grounds. Study A's euthanasia schedule is on days 1, 2, 3, 4, 7, 14 and 21 with 5 subjects each day. Study B's schedule is on days 8 and 28 with 5 and 10 subjects, respectively. Tissues collected for Study A include stomach, diaphragm and intestine. Study B tissues include brain, lung, liver and kidney. Tissues are processed for HE and IHC (T. gondii antibody).

Navigate back to Figure 1.

## Figure 2 Long description

The image A showing a pink and purple micrograph with a large black oval outline around a central area containing many small dark dots; label A at top left; scale bar text “100 µm” at bottom right. The image B showing a pink and purple micrograph with a dotted curved line near the left edge and a small black circle near the center-left; label B at top left; scale bar text “100 µm” at bottom right. The image C showing a pale blue micrograph with a single dark arrow pointing to a brown circular spot near the center-right; label C at top left; scale bar text “50 µm” at bottom right. The image D showing a pale blue micrograph with two dark arrows pointing toward multiple small yellow-brown dots scattered across the field; label D at top left; scale bar text “100 µm” at bottom right. The image E showing a pale blue micrograph with three dark arrows pointing toward multiple small yellow-brown dots, including a larger yellow-brown spot near the right side; label E at top left; scale bar text “100 µm” at bottom right. The image F showing a pink and purple micrograph with one dark arrow pointing to a small cluster of red dots near the center; label F at top left; scale bar text “50 µm” at bottom right.

Navigate back to Figure 2.

## Figure 3 Long description

The image A showing a pink and purple tissue section with three black oval outlines and one smaller irregular outline near the center. A larger outlined area at the lower right contains a pale oval space with red material inside. A scale bar at the lower right reads 200 µm. The image B showing a densely packed purple cellular field with several pale spaces. Four black arrows point toward small areas within the tissue. A scale bar at the lower right reads 100 µm. The image C showing a pink background with many small round clear spaces and scattered purple nuclei. An irregular region is enclosed by a dotted black outline, with a smaller dotted outline inside it. A scale bar at the lower right reads 100 µm. The image D showing a pale blue background with faint cellular shapes. Two black arrows point toward a small brown spot near the center. A scale bar at the lower left reads 50 µm. The image E showing a pale blue tissue section with many small clear spaces and scattered purple nuclei. Two black circles outline areas containing brown staining. Several black arrows point toward small brown spots in different areas. A scale bar at the lower right reads 100 µm. The image F showing a pale blue tissue section with scattered purple nuclei and multiple small brown spots. Two thick black arrows point toward brown spots near the center-left. One thin black arrow points toward a brown spot near the lower right. A scale bar at the lower right reads 100 µm.

Navigate back to Figure 3.

## Figure 4 Long description

The image A showing a pink and purple stained tissue section with elongated, curved bands. A dashed black outline follows a long, narrow region near the center. A scale bar at the bottom reads 100 micrometers. The image B showing a low-magnification pink and purple stained tissue section with a broad, rounded tissue area. A rectangular inset is placed at the lower left of the tissue. A scale bar at the bottom reads 200 micrometers. The image C showing pink and purple stained tissue with multiple rounded and tubular profiles near the top and a densely cellular area below. A dashed black outline encloses an irregular region on the right half. Two small black arrowheads point toward small dark features near the lower left of the outlined region. A scale bar at the bottom reads 200 micrometers. The image D showing pink and purple stained tissue with many polygonal spaces separated by thin septa. Several black arrowheads point to small dark features in the upper half. A dashed black outline encloses a small irregular area near the lower left. A scale bar at the bottom reads 100 micrometers. The image E showing a pale blue to gray stained tissue section with scattered light brown punctate staining. A rectangular inset is placed at the lower left. A black arrow points upward near the bottom center. A scale bar at the bottom reads 200 micrometers. The image F showing a pale blue to gray stained tissue section with scattered light brown punctate staining. A rectangular inset is placed at the lower left. A black arrow points upward near the bottom center. A scale bar at the bottom reads 200 micrometers.

Navigate back to Figure 4.

## Figure 5 Long description

The image contains two nonlinear PCA biplots labeled A and B. Image A shows the brain data with Dim1 accounting for 66.6 percent and Dim2 for 21.6 percent of variance. Key clusters are observed for isolates A3 and A4, with A4 showing higher scores. Arrows indicate variables like NF.Size and GF.Size, with tachyzoites associated with A3 and pseudocysts with A4. Yes/No labels indicate meningitis levels. Image B shows peripheral organ data with Dim1 at 57.3 percent and Dim2 at 17.4 percent. Clusters for A3 and A4 are visible, with A3 having higher scores. Arrows represent variables like S.NF and St.Tg.T, with high parasite loads in A3. The Yes/No labels and marker shapes differentiate isolates. The biplots highlight clustering differences between brain and peripheral organs, with stronger separation in the brain. Key vectors like NF.Size and S.NF are most influential, indicating variable loadings on Dim1 and Dim2.

Navigate back to Figure 5.

## Figure 6 Long description

The image A showing a purple-stained tissue section with many small dark nuclei on a light purple background. A black circle encloses a dense cluster of dark nuclei near the center. A scale bar at the bottom right reads 100 micrometers. The image B showing a purple-stained tissue section with scattered small dark nuclei on a light purple background. A black oval encloses a lighter central area with fewer nuclei than the surrounding field. A black arrow near the bottom points toward the right. A scale bar at the bottom right reads 200 micrometers. The image C showing a purple-stained tissue section with a large pale area and a darker purple region toward the right side. A vertical bracket-like marking runs along the left edge. A black rectangle marks a region in the lower-left area of the tissue field. A scale bar at the bottom right reads 500 micrometers. The image D showing a pale blue tissue section with scattered small dark nuclei. Several small brown-stained spots are present. Three black arrows point to separate brown-stained spots, with one arrow near the left side, one near the upper middle and one near the right side. A scale bar at the bottom right reads 100 micrometers. The image E showing a pale blue tissue section with scattered small dark nuclei and a few small brown-stained spots. Three black arrows point to three separate brown-stained spots across the upper half of the field. A scale bar at the bottom right reads 200 micrometers. The image F showing a pale blue tissue section with scattered small dark nuclei and a few small brown-stained spots. Three black arrows point to separate brown-stained spots, with two arrows on the left side and one arrow on the right side. A scale bar at the bottom right reads 100 micrometers.

Navigate back to Figure 6.

## Figure 7 Long description

The image A showing a purple-stained tissue section with a pale background. A thick black arrow points to the upper right edge. Three small black arrowheads point to structures along the left-to-right slanted tissue edge. A scale bar at the lower right reads 100 micrometers. The image B showing a purple-stained tissue section with multiple rounded and tubular profiles. Two black circles mark separate areas, one near the upper center and one near the lower left. A scale bar at the lower right reads 100 micrometers. The image C showing a purple-stained tissue section with multiple rounded and tubular profiles. One black circle marks an area near the lower right. A scale bar at the lower right reads 100 micrometers. The image D showing a lightly stained tissue section with several brown-stained spots near the upper left slanted edge. Three black arrows point to brown-stained areas along the edge. A scale bar at the lower right reads 100 micrometers. The image E showing a lightly stained tissue section with many clear round spaces and thin purple-stained boundaries. A black arrow points to a small brown-stained spot near the center. A scale bar at the lower right reads 50 micrometers. The image F showing a lightly stained tissue section with clustered polygonal profiles and pale purple staining. A black arrow points to a small brown-stained spot near the center-left. A scale bar at the lower right reads 50 micrometers.

Navigate back to Figure 7.

## Figure 8 Long description

The image A showing pink and purple stained tissue with many open spaces and thin septa. A black oval outline encloses a denser cluster of tissue in the center. A scale bar at the bottom right reads 50 micrometers. The image B showing pink and purple stained tissue with a large open space at the top and denser cellular areas below. Three black arrowheads mark locations near the upper open space and within the tissue. A scale bar at the bottom right reads 100 micrometers. The image C showing pink and purple stained tissue with multiple open spaces separated by thin septa and a denser band of tissue running diagonally through the center. A scale bar at the bottom right reads 100 micrometers. The image D showing pale blue and gray stained tissue with many open spaces and thin septa. One black arrow points to a small darker area near the center. A scale bar at the bottom right reads 50 micrometers. The image E showing pale blue and gray stained tissue with scattered brown stained cells. Three black arrows point to brown stained cells in the upper half and lower right area. A scale bar at the bottom right reads 50 micrometers. The image F showing pink and purple stained tissue with a large curved open lumen-like space along the upper left and a thick surrounding tissue wall. A dotted black line traces part of the inner border of the wall. A scale bar at the bottom right reads 200 micrometers.

Navigate back to Figure 8.

## Figure 9 Long description

The image consists of two scatter plots labeled A and B. Plot A represents the nonlinear PCA biplot for brain samples, while plot B represents peripheral organ samples from Study B. In plot A, the horizontal axis is labeled Dim1 with a percentage of 60.3 and the vertical axis is labeled Dim2 with a percentage of 22.6. The plot shows data points for groups B1 through B8. Groups B2, B3, B4, B6 and B7 are concentrated on the right side of the vertical axis, indicating higher pathology and presence compared to B5 and B8, which are mostly on the left. Group B1 is around zero on each side of the axis, indicating mild to moderate pathology. Notable outliers from B2 and B3 are present on the right, reflecting the presence of NF. In plot B, the horizontal axis is labeled Dim1 with a percentage of 44.9 and the vertical axis is labeled Dim2 with a percentage of 18.1. The plot shows a different distribution where isolates that dominated the right side of the brain plot (B2, B3, B4 and B7) are found on the left side of the peripheral organ plot. Conversely, isolates from the left side of the brain plot (B5, B6 and B8) appear more on the right side of the peripheral organ plot. Group B1 is associated with low lesion and parasite scores and is positioned on the left of the axis. The legend indicates different symbols and colors for each group, aiding in the identification of data clusters and distribution patterns.

Navigate back to Figure 9.

## Figure 10 Long description

Panel 1 shows Study A vs Study B for genotype 3 in the brain. The x-axis is Dim1 (88.5 percent) and the y-axis is Dim2 (8.6 percent). Black squares represent data points. Key vectors include Tg.T and Tg.P, indicating strong influence on Dim1. Panel 2 shows genotype 3 in peripheral organs. The x-axis is Dim1 (43.3 percent) and the y-axis is Dim2 (19.8 percent). Vectors like Lu.NF and Li.NF are prominent, showing separation between studies. Panel 3 shows genotype 6 in the brain. The x-axis is Dim1 (63.9 percent) and the y-axis is Dim2 (21.6 percent). Triangles and squares represent data points, with vectors like Tg.T and PC.Size indicating strong Dim1 influence. Panel 4 shows genotype 6 in peripheral organs. The x-axis is Dim1 (72.1 percent) and the y-axis is Dim2 (8.9 percent). Vectors such as Ki.NF and Lu.NF are significant. Overall, genotype 3 shows more separation in peripheral organs, while genotype 6 shows more influence in the brain. The plots highlight differences in pathology and parasite burden between studies and genotypes.

Navigate back to Figure 10.

## Table 3 Long description

Counts are given as animals reaching each scheduled euthanasia time point (days 1, 2, 3, 4, 7, 14, and 21 post‑infection) for groups A1–A4. A2 and A4 each recorded 5 of 5 animals at every listed time point from day 1 through day 21. A3 matched A2/A4 through day 7 (5/5 at days 1, 2, 3, 4, and 7) but dropped to 2/5 at day 14 and 0/5 at day 21, indicating animals did not remain to those scheduled endpoints. A1 has data only at day 21, where 5/5 reached the scheduled time point; earlier days are not applicable/recorded. The reduced A3 counts at days 14 and 21 reflect early euthanasia on welfare grounds, so these values should not be interpreted as missing data or noncompliance with scheduling.

Navigate back to Table 3.

## Table 4 Long description

The table reports, for Study B groups B1–B8 (15 animals per group), how many animals reached scheduled euthanasia time points at day 8 and day 28 post-infection. At day 8, every group reached the target of 5 animals euthanised (shown as 5/5). At day 28, outcomes differed: B1 had 10 animals euthanised at day 28, B7 had 8/10, and B2–B4 had 5/10, 6/10, and 7/10 respectively. In contrast, none of the remaining 10 animals in groups B5, B6, or B8 reached the day 28 time point (0/10 in each). The day-28 values expressed as “x/10” indicate how many of the 10 animals intended for day 28 actually reached that scheduled endpoint. Some animals were euthanised earlier for welfare reasons, which reduces the number available to reach day 28 and should be considered when comparing groups.

Navigate back to Table 4.

## Table 5 Long description

The table compares ToxoDB RFLP genotypes across two studies by overall pathogenicity, the ranked organs affected (most to least), and the dominant parasite stage. In both studies, genotype #6 is classified as severe and dominated by tachyzoites; organs include liver first in both, with Study A listing liver, intestine, lung, kidney, diaphragm, brain, stomach and Study B listing liver, lung, kidney, brain. Study B reports additional severe disease for genotype #13 (lung then liver, kidney, brain) with tachyzoites, while Study A has no data for #13. Moderate pathogenicity appears for genotype #8 in Study A (lung then brain, liver, intestine, diaphragm, kidney, stomach) with pseudocysts, and for genotypes #141, #265, and #3 in Study B, typically involving brain and lung plus liver and kidney. For #141, #265, and #3 in Study B, parasite stage differs by site: pseudocysts in brain and tachyzoites in peripheral organs. Mild disease is reported for genotype #3 in Study A (liver and intestine; parasite not observed) and for genotype #1 in Study B (brain, lung, liver; pseudocysts). Dashes indicate genotypes not reported in a given study, so cross-study comparisons are limited to genotypes with entries in both studies.

Navigate back to Table 5.
