# Supplementary figures and images for: Pathology and parasite distribution in mice challenged with Toxoplasma gondii from different geographical origins
Source: Parasitology. 2026 Jan 15;153(3):386–402. doi: 10.1017/S0031182026101589 (PMC13215732; doi:10.1017/S0031182026101589)

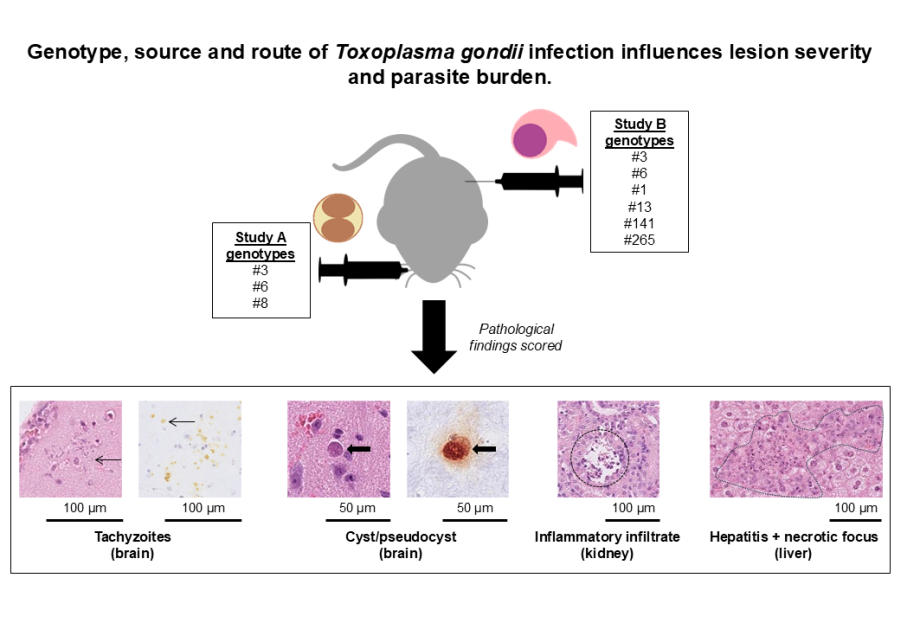

Supplement: Black et al. supplementary material 1 — Black et al. supplementary material [file S0031182026101589sup001.png]
