# Supplementary material for: Pathology and parasite distribution in mice challenged with Toxoplasma gondii from different geographical origins
Source: Parasitology. 2026 Jan 15;153(3):386–402. doi: 10.1017/S0031182026101589 (PMC13215732; doi:10.1017/S0031182026101589)
Supplement: Black et al. supplementary material 2 — Black et al. supplementary material [file S0031182026101589sup002.pdf]

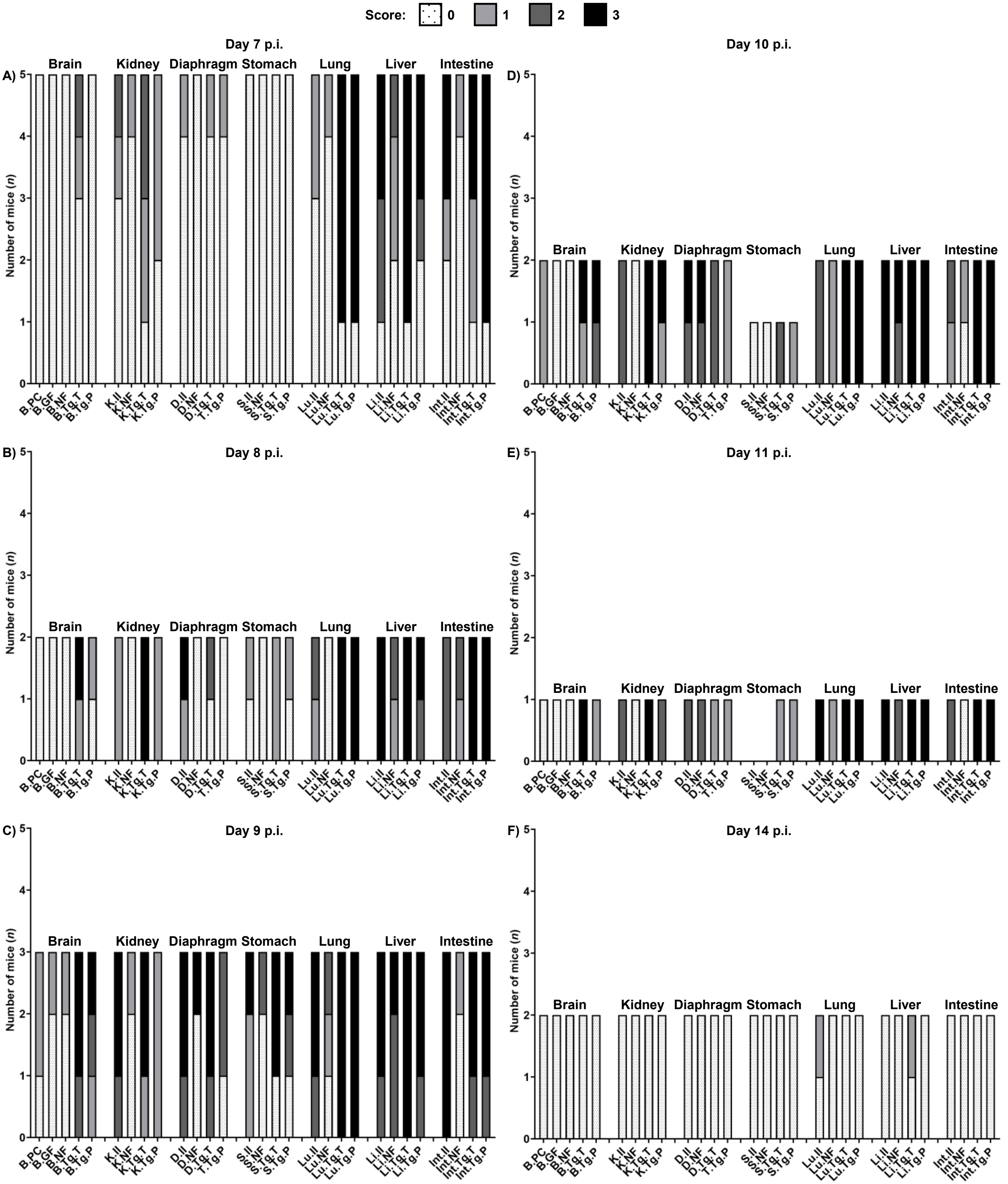

S1 Fig. Summary of significant pathological changes and parasite distribution for A3 by day. The graphs summarise the scores given for the severity of lesions (II; inflammatory infiltrate, NF; necrotic foci) and *T. gondii* life stage abundance in the peripheral organs of mice euthanised at day 7 **(A)**, 8 **(B)**, 9 **(C)**, 10 **(D)**, 11 **(E)** and 14 **(F)** p.i. Scores (0 to 3) are represented by a different colour. All mice scheduled to be euthanised at day 7 p.i. were present (*n*= 5), however many mice scheduled to be euthanised at day 14 or 21 p.i. (*n*= 10) were euthanised between day 8 and 14 p.i. due to clinical signs of toxoplasmosis. Some stomach samples were not available for assessment (days 10 and 11 p.i.).

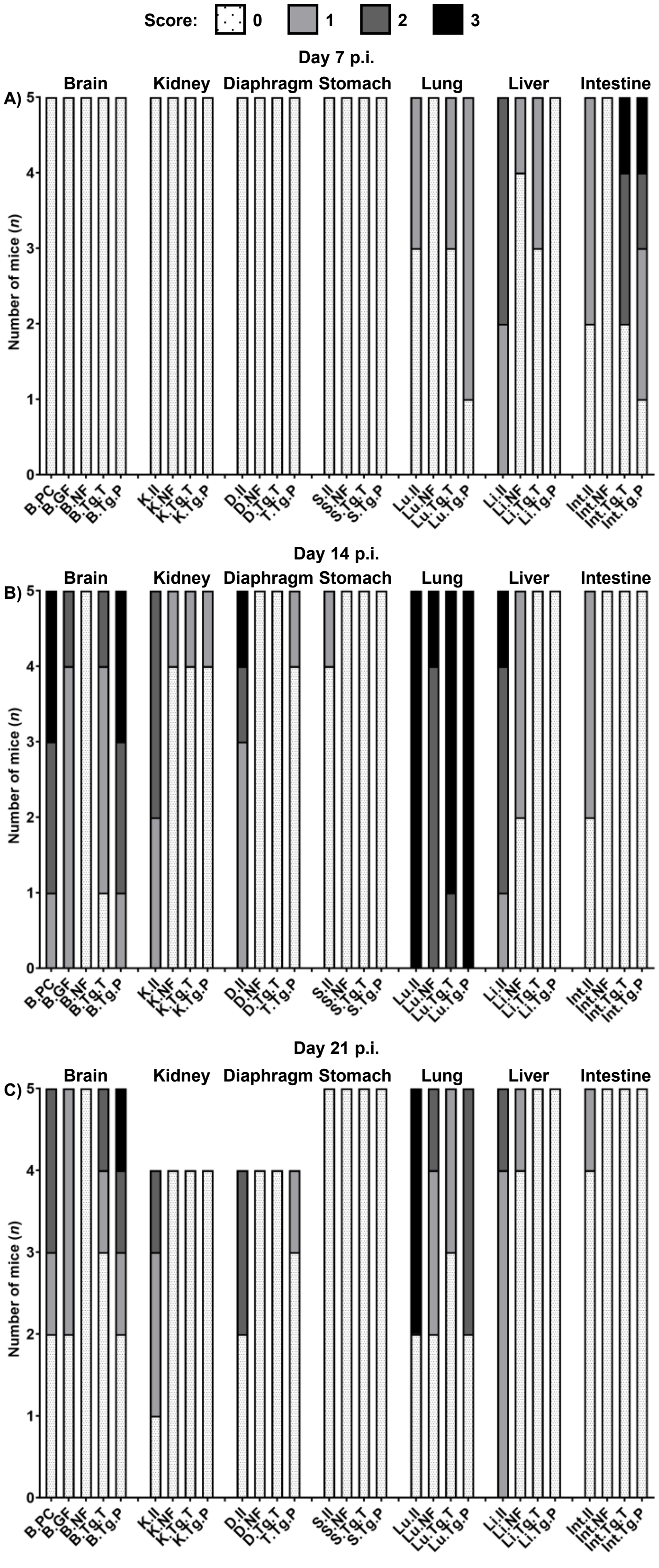

S2 Fig. Summary of significant pathological changes and parasite distribution for A4 by day. The graphs were used to summarise the lesion and parasite scores for peripheral organs at days 7 (A), 14 (B) and 21 (C) p.i. Scores (0 to 3) are represented by a different colour. Kidney and diaphragm sections were not available for one animal at day 21 p.i.

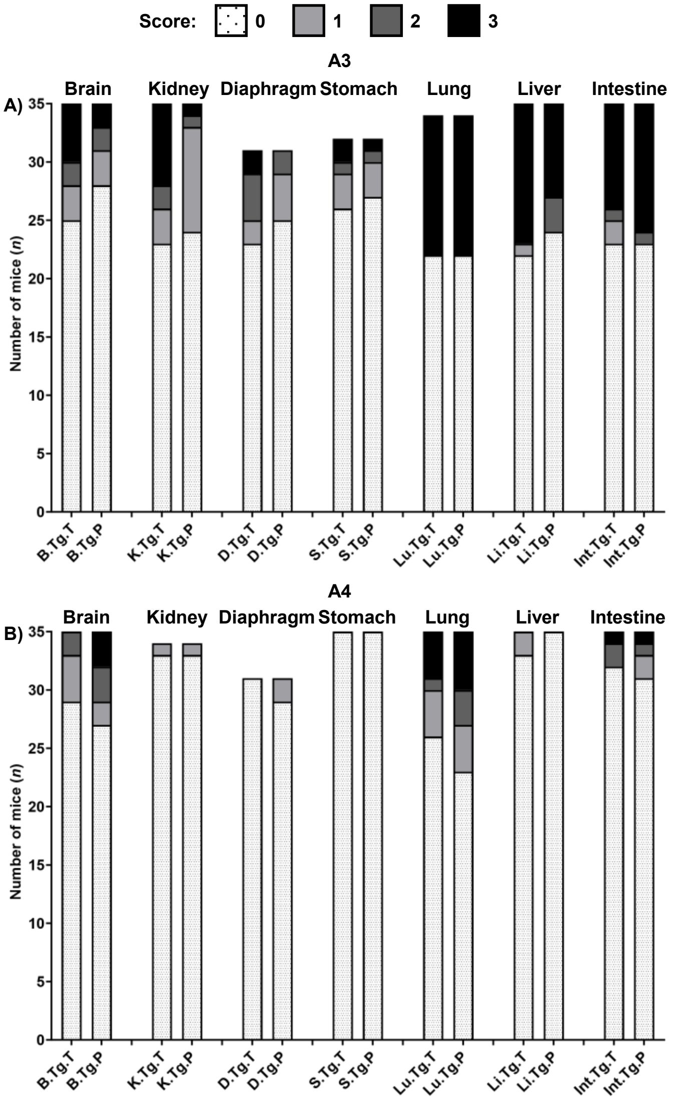

S3 Fig. Assessment of *T. gondii* stage found in tissues of mice in groups A3 and A4. In **A)** and **B)**, the scores for *T. gondii* tachyzoite (T) and cyst (C) abundance are plotted for all organs belonging to groups A3 **(C)** and A4 **(D)** from all days of the experiment. The parasite was not identified in the negative control group (A1) or A2. Some sections were not available for analysis. The scores are differentiated by colour.

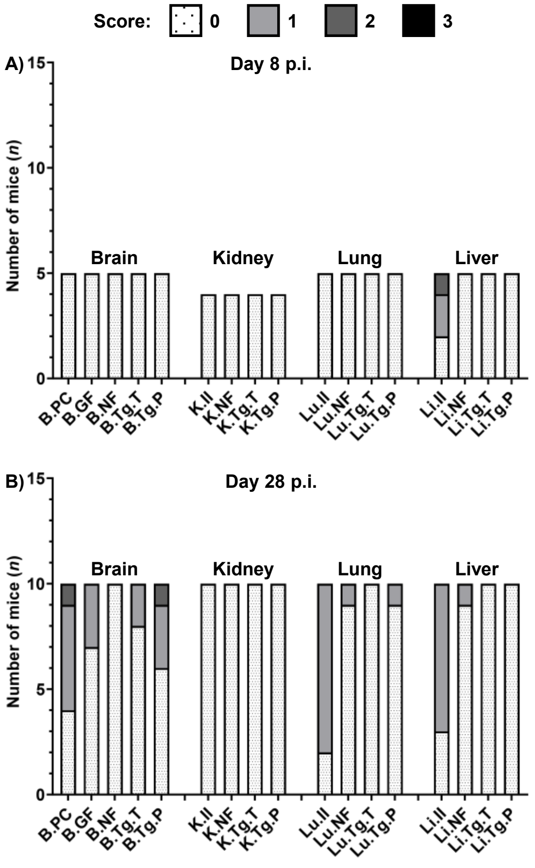

S4 Fig. Summary of significant pathological changes and parasite distribution for B1 by day. The graphs summarise the scores given for the severity of lesions (II; inflammatory infiltrate, NF; necrotic foci) and *T. gondii* life stage abundance in the peripheral organs of mice euthanised at day 7 **(A)** and 28 **(B)** p.i. Scores (0 to 3) are represented by a different colour. All mice scheduled to be euthanised at day 8 p.i. (*n*= 5) and day 28 p.i. (*n*= 10) were present. A kidney sample was unavailable at day 8 p.i.

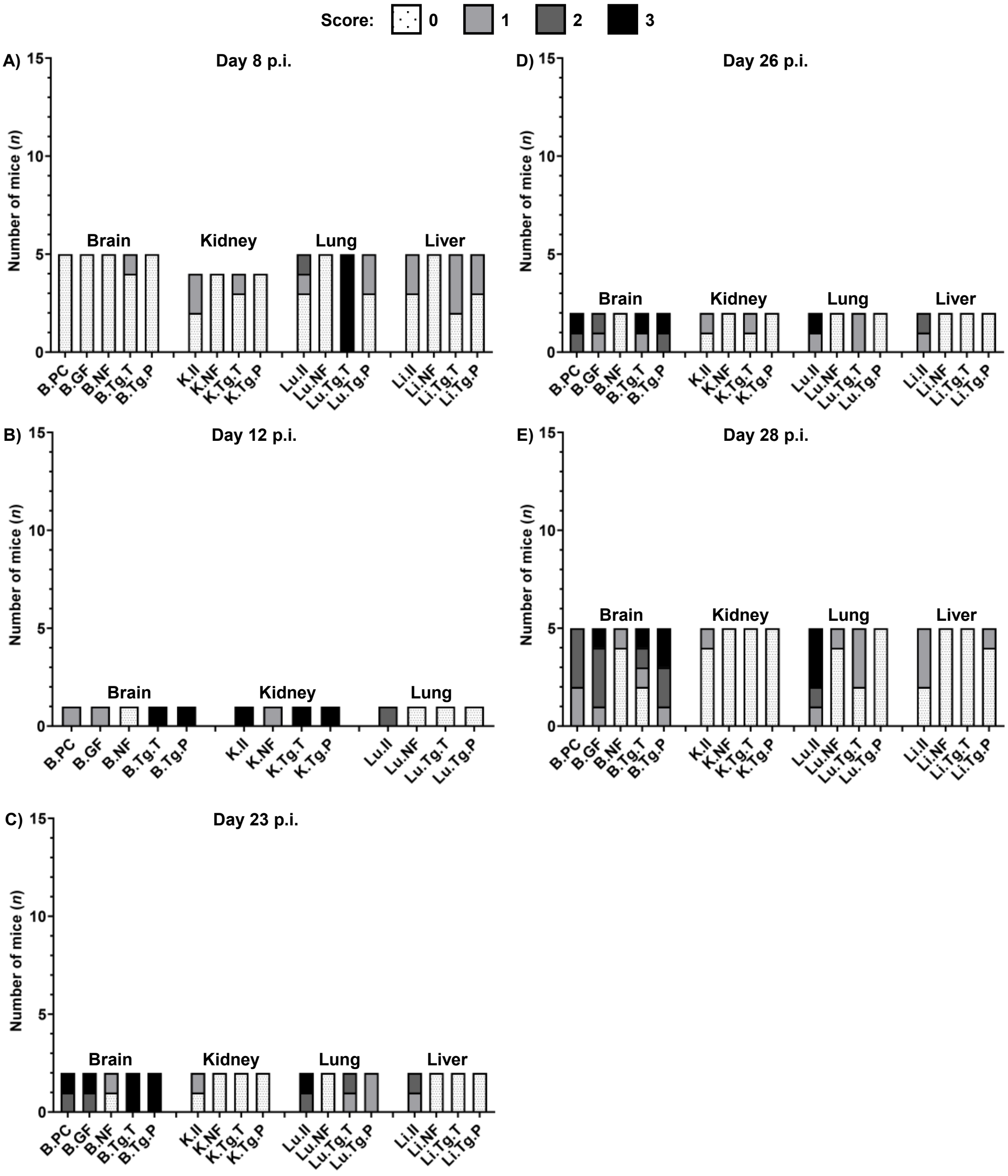

S5 Fig. Summary of significant pathological changes and parasite distribution for B2 by day. The graphs summarise the scores given for the severity of lesions (II; inflammatory infiltrate, NF; necrotic foci) and *T. gondii* life stage abundance in the organs of mice euthanised at day 8 **(A)**, 12 **(B)**, 23 **(C)**, 26 **(D)** and 28 **(E)** p.i. Scores (0 to 3) are represented by a different colour. All mice scheduled to be euthanised at day 8 p.i. (*n*= 5) were present and mice in the survival experiment were euthanised at days 12 (*n*= 1), 23 (*n*= 2) and 26 (*n*= 2) due to clinical signs associated with toxoplasmosis, and 28 (*n*= 5) p.i., the endpoint of the experiment. A kidney and liver sample was unavailable at day 8 and 12 p.i. respectively.

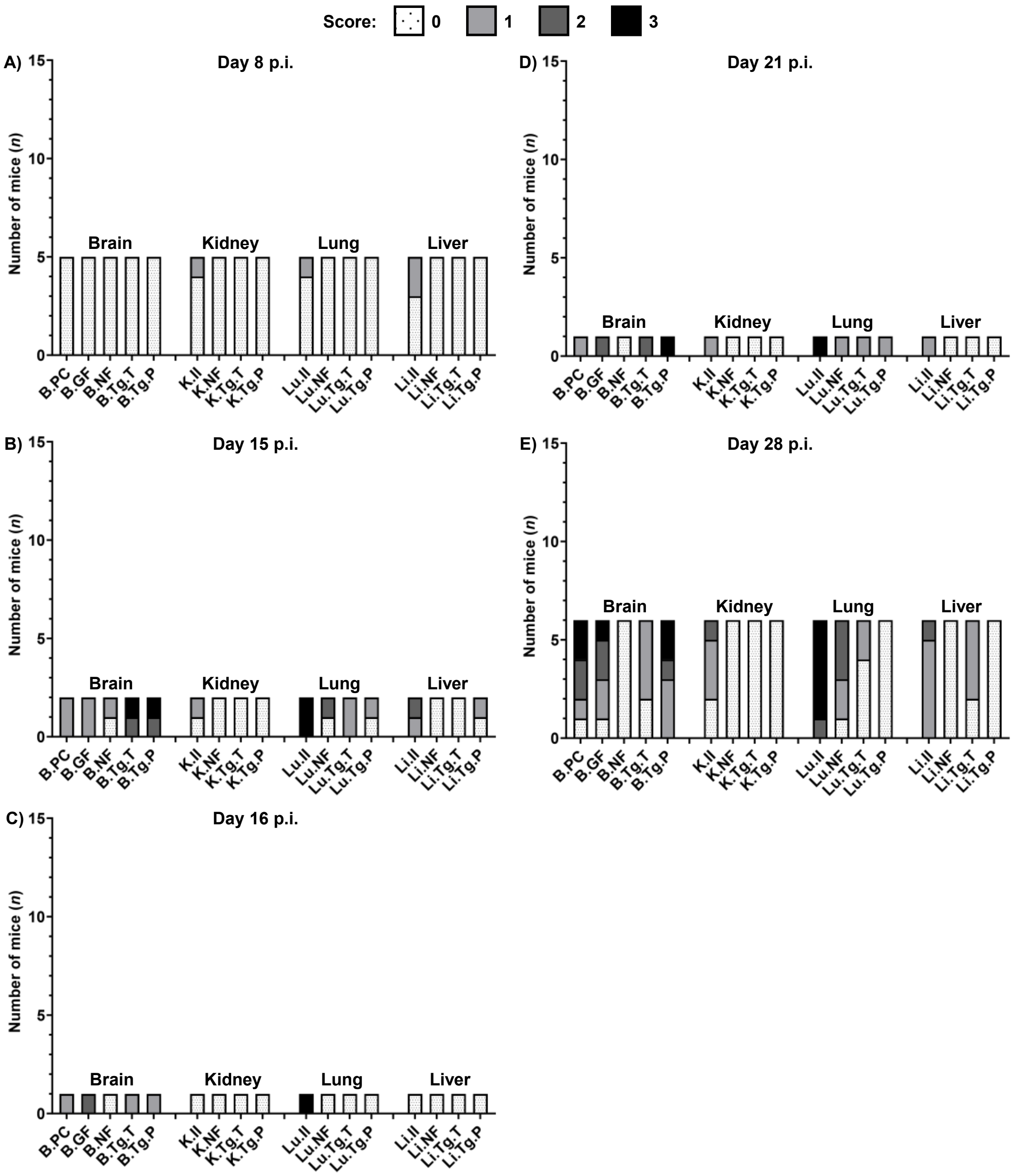

S6 Fig. Summary of significant pathological changes and parasite distribution for B3 by day. The graphs summarise the scores given for the severity of lesions (II; inflammatory infiltrate, NF; necrotic foci) and *T. gondii* life stage abundance in the organs of mice euthanised at day 8 **(A)**, 15 **(B)**, 16 **(C)**, 21 **(D)** and 28 **(E)** p.i. Scores (0 to 3) are represented by a different colour. All mice scheduled to be euthanised at day 8 p.i. (*n*= 5) were present and mice in the survival experiment were euthanised at days 15 (*n*= 2), 16 (*n*= 1) and 21 (*n*= 1) due to clinical signs associated with toxoplasmosis, and the scheduled endpoint, 28 (*n*= 6) p.i.

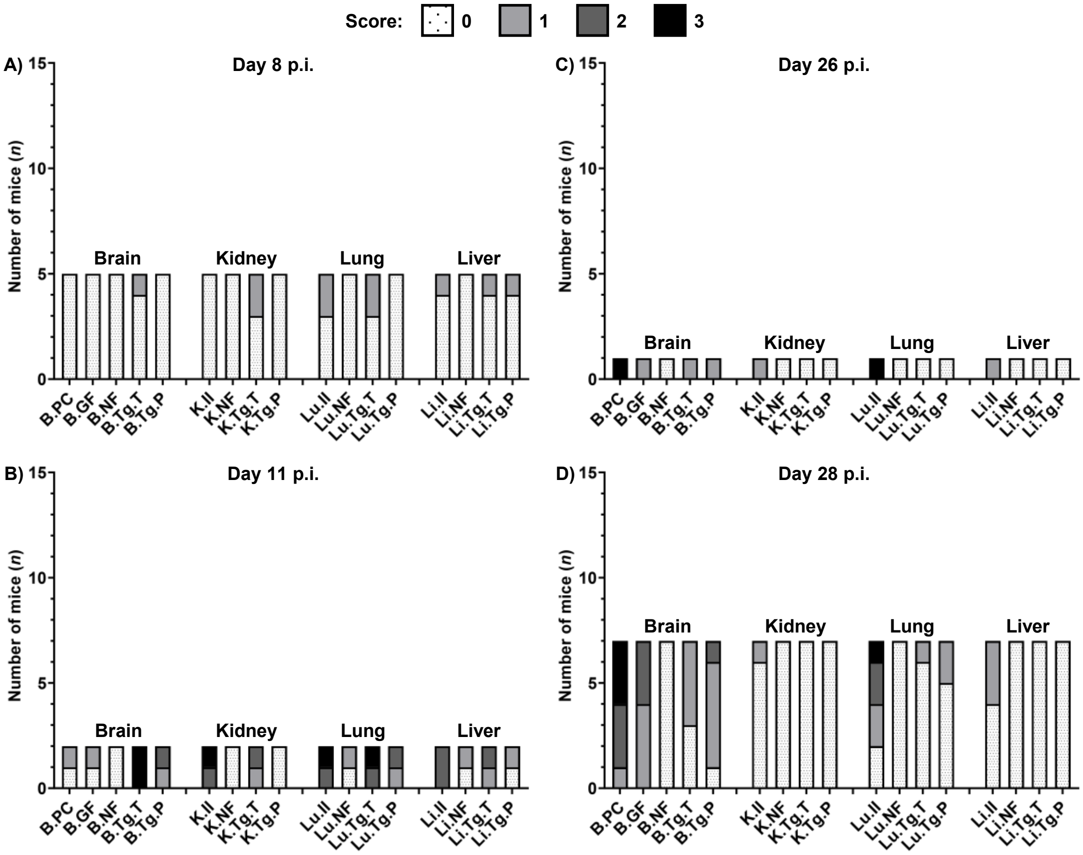

S7 Fig. Summary of significant pathological changes and parasite distribution for B4 by day. The graphs summarise the scores given for the severity of lesions (II; inflammatory infiltrate, NF; necrotic foci) and *T. gondii* life stage abundance in the organs of mice euthanised at day 8 **(A)**, 11 **(B)**, 26 **(C)** and 28 **(D)** p.i. Scores (0 to 3) are represented by a different colour. All mice scheduled to be euthanised at day 8 p.i. (*n*= 5) were present and mice in the survival experiment were euthanised at days 11 (*n*= 2) and 26 (*n*= 1) due to clinical signs associated with toxoplasmosis, and 28 (*n*= 7) p.i., the last scheduled timepoint.

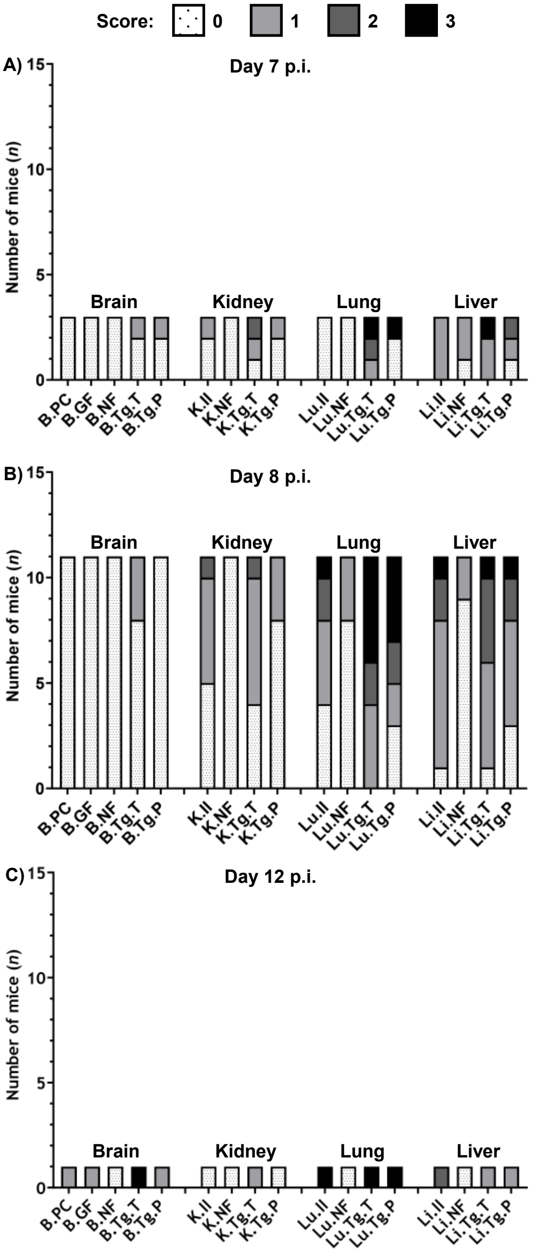

S8 Fig. Summary of significant pathological changes and parasite distribution for B5 by day. The graphs summarise the scores given for the severity of lesions (II; inflammatory infiltrate, NF; necrotic foci) and *T. gondii* life stage abundance in the organs of mice euthanised at day 7 **(A)**, 8 **(B)** and 12 **(C)** p.i. Scores (0 to 3) are represented by a different colour. Mice were euthanised at day 7 (*n*= 3), day 8 (*n*= 11) and 12 (*n*= 1) p.i. due to clinical signs associated with toxoplasmosis.

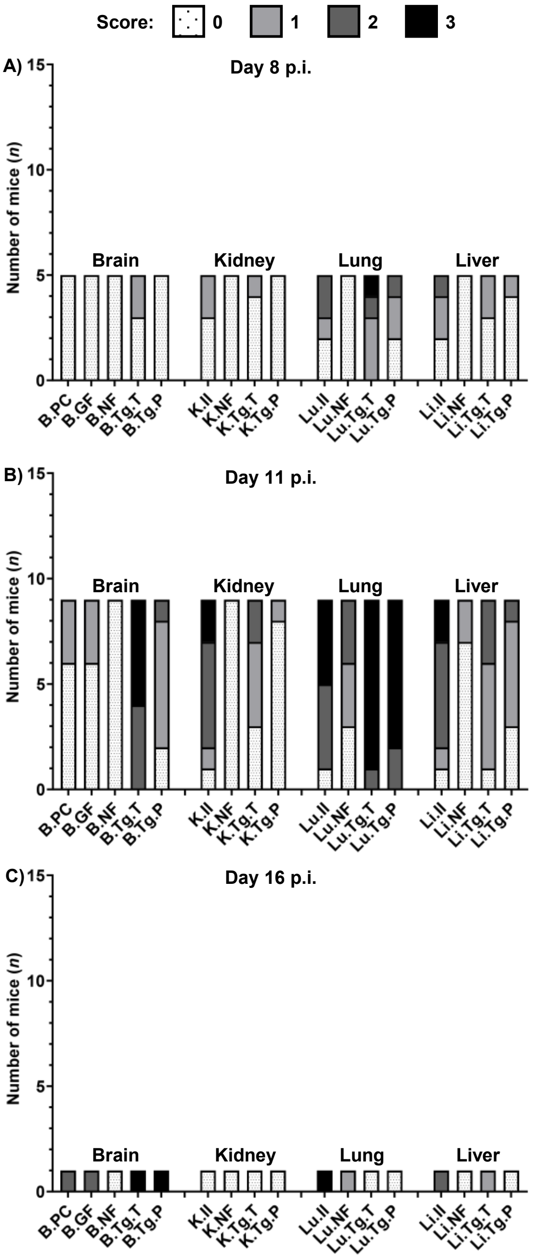

S9 Fig. Summary of significant pathological changes and parasite distribution for B6 by day. The graphs summarise the scores given for the severity of lesions (II; inflammatory infiltrate, NF; necrotic foci) and *T. gondii* life stage abundance in the organs of mice euthanised at day 8 **(A)**, 11 **(B)** and 16 **(C)** p.i. Scores (0 to 3) are represented by a different colour. All mice scheduled for euthanasia at day 8 p.i. (*n*= 5) were present and mice in the survival experiment were euthanised at day 11 (*n*= 9) and 16 (*n*= 1) p.i. due to clinical signs associated with toxoplasmosis.

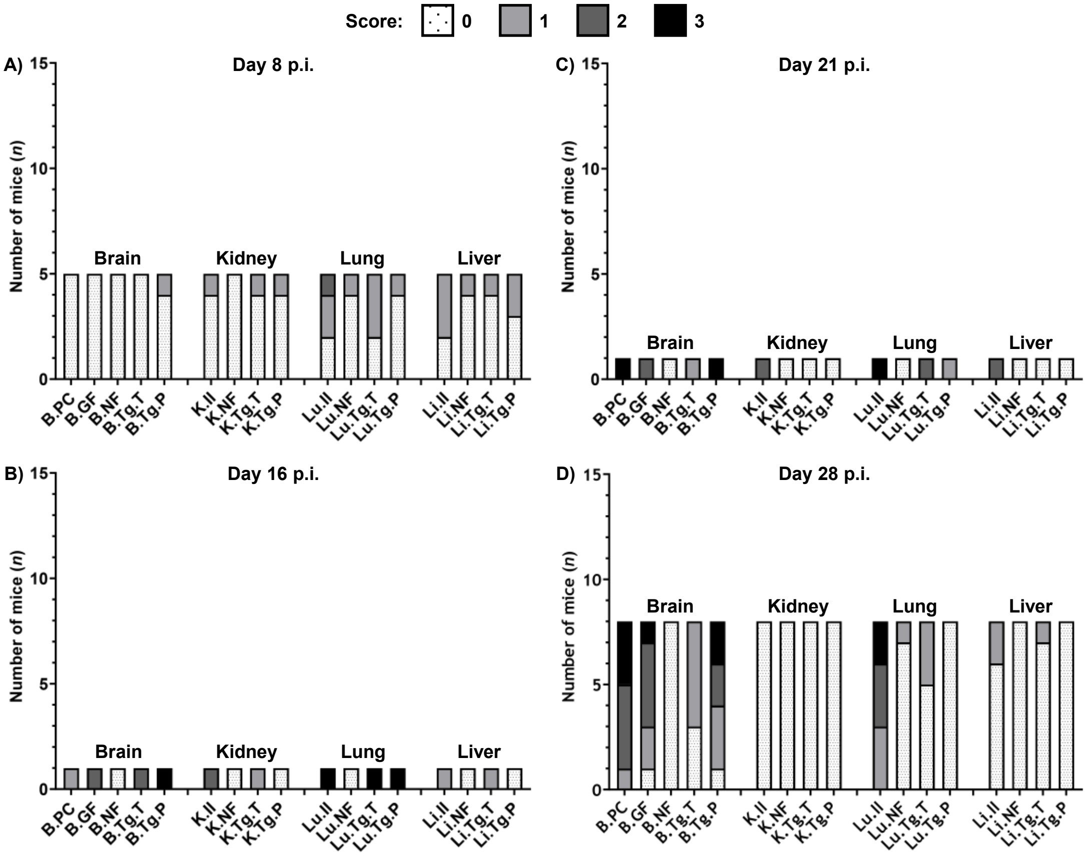

S10 Fig. Summary of significant pathological changes and parasite distribution for B7 by day. The graphs summarise the scores given for the severity of lesions (II; inflammatory infiltrate, NF; necrotic foci) and *T. gondii* life stage abundance in the organs of mice euthanised at day 8 **(A)**, 16 **(B)**, 21 **(C)** and 28 **(D)** p.i. Scores (0 to 3) are represented by a different colour. All mice scheduled to be euthanised at day 8 p.i. (*n*= 5) were present and mice in the survival experiment were euthanised at days 16 (*n*= 1) and 21 (*n*= 1) p.i. due to clinical signs associated with toxoplasmosis, and 28 (*n*= 8) p.i., the endpoint of the experiment.

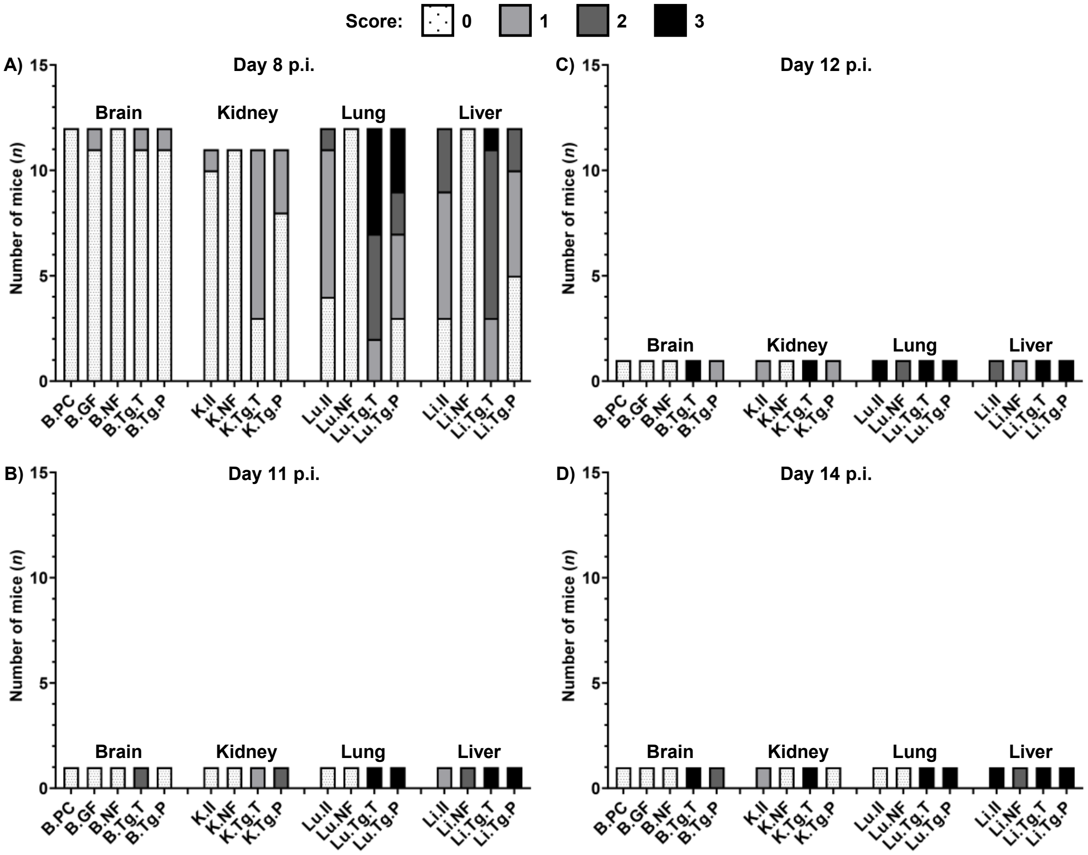

S11 Fig. Summary of significant pathological changes and parasite distribution for B8 by day. The graphs summarise the scores given for the severity of lesions (II; inflammatory infiltrate, NF; necrotic foci) and *T. gondii* life stage abundance in the organs of mice euthanised at day 8 **(A)**, 11 **(B)**, 12 **(C)** and 14 **(D)** p.i. Scores (0 to 3) are represented by a different colour. Mice were euthanised at day 8 p.i. (*n*= 12), 11 (*n*= 1), 12 (*n*= 1) and 14 (*n*= 1) p.i. due to clinical signs associated with toxoplasmosis.

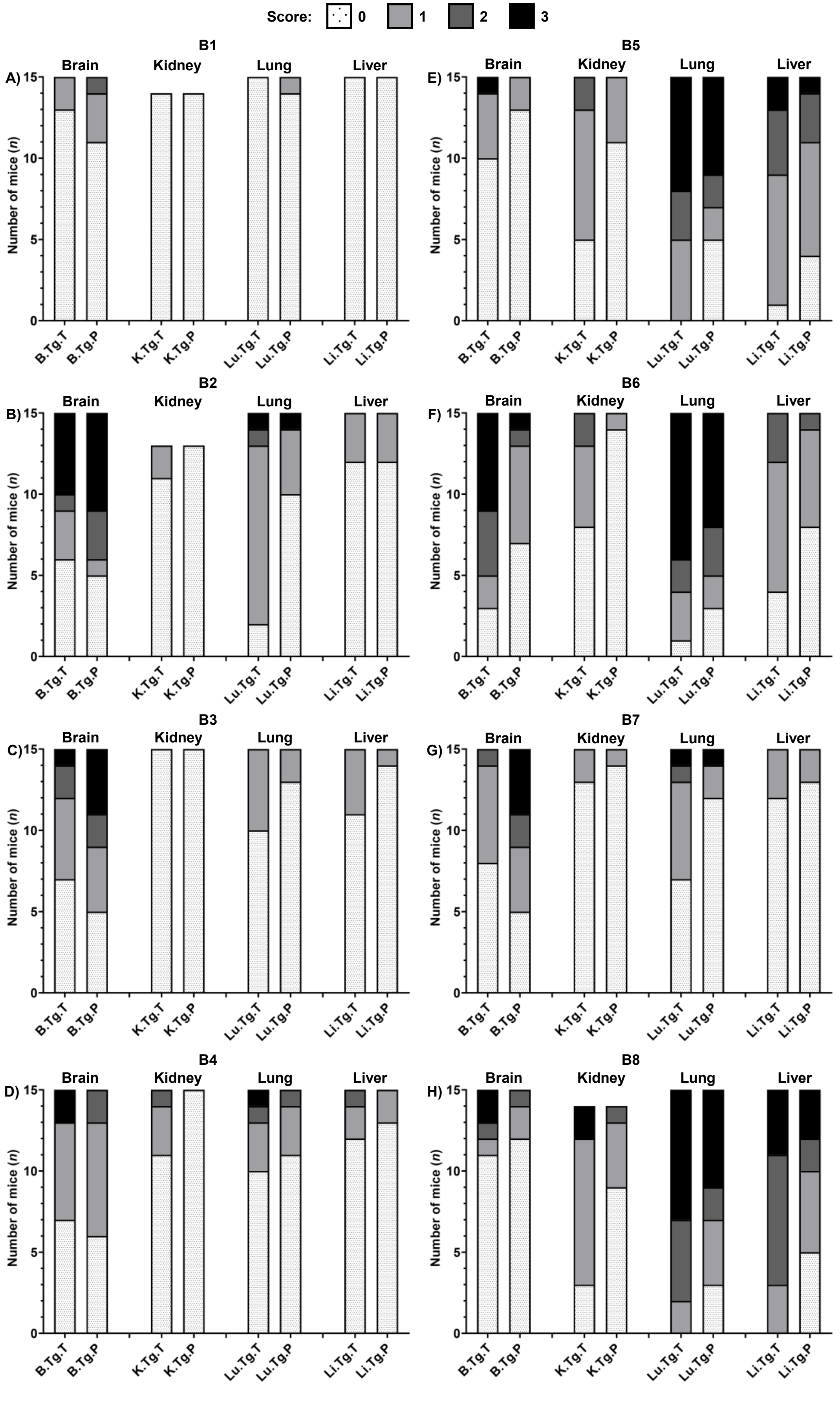

S12 Fig. Assessment of *T. gondii* stage found in tissues of mice in groups B1 to B8. The scores for *T. gondii* tachyzoite (T) and pseudocyst (P) abundance are plotted for all organs belonging to groups B1 (A), B2 (B), B3 (C), B4 (D), B5 (E), B6 (F), B7 (G) and B8 (H) from all days of the experiment. Some kidney sections were not available for analysis from B1, B2 and B8. The scores are differentiated by colour.
